# Trojan Horse Delivery of 4,4′‐Dimethoxychalcone for Parkinsonian Neuroprotection

**DOI:** 10.1002/advs.202004555

**Published:** 2021-03-03

**Authors:** Wenlong Zhang, Huaqing Chen, Liuyan Ding, Junwei Gong, Mengran Zhang, Wenyuan Guo, Pingyi Xu, Shiying Li, Yunlong Zhang

**Affiliations:** ^1^ Department of Neurology The First Affiliated Hospital of Guangzhou Medical University Guangzhou 510120 China; ^2^ Key Laboratory of Molecular Target & Clinical Pharmacology and the State Key Laboratory of Respiratory Disease School of Pharmaceutical Sciences & The Fifth Affiliated Hospital Guangzhou Medical University Guangzhou 511436 China; ^3^ Key Laboratory of Neurological Function and Health School of Basic Medical Sciences Guangzhou Medical University Guangzhou 511436 China

**Keywords:** blood brain barrier, brain targeted delivery, DMC, neuroinflammation, oxidative stress, TH ubiquitination

## Abstract

Parkinson's disease (PD) is characterized by the progressive deterioration of dopamine (DA) neurons, and therapeutic endeavors are aimed at preventing DA loss. However, lack of effective brain delivery approaches limits this strategy. In this study, a “Trojan horse” system is used for substantia nigra‐targeted delivery of a blood brain barrier‐penetrating peptide (RVG29) conjugated to the surface of nanoparticles loaded with the natural autophagy inducer 4,4′‐dimethoxychalcone (DMC) (designated as RVG‐nDMC). Here, the neuroprotective effects of DMC are demonstrated in PD. Specifically, RVG‐nDMC penetrates the blood brain barrier with enhanced brain‐targeted delivery efficiency and is internalized by DA neurons and microglia. In vivo studies demonstrate that RVG‐nDMC ameliorates motor deficits and nigral DA neuron death in PD mice without causing overt adverse effects in the brain or other major organs. Moreover, RVG‐nDMC reverses tyrosine hydroxylase ubiquitination and degradation, alleviates oxidative stress in DA neurons, and exerts antiinflammatory effects in microglia. The “Trojan horse” strategy for targeted delivery of DMC thus provides a potentially powerful and clinically feasible approach for PD intervention.

## Graphical abstract

A Trojan horse system (RVG‐nDMC) is constructed for blood brain barrier‐penetrated delivery of 4,4′‐dimethoxychalcone (DMC) to ameliorate motor deficits and nigral dopamine (DA) neuronal death in Parkinson's disease. Mechanistically, RVG‐nDMC reduces tyrosine hydroxylase ubiquitination and degradation, alleviates the oxidative stress in DA neurons, and exerts antiinflammation effects in microglia for synergistic Parkinsonian intervention.

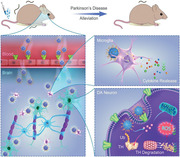

## 1 Introduction

Parkinson's disease (PD) is the most prevalent neurodegenerative movement disorder, affecting ≈2–3% of the population ≥65 years of age worldwide.^[^ ^1^, ^2^ ^]^ Pathologically, PD is characterized by the loss of dopamine (DA) neurons in the pars compacta of the substantia nigra (SNpc) and the formation of intracytoplasmic Lewy bodies due to accumulation of misfolded *α*‐synuclein.^[^ ^3^, ^4^ ^]^ The progressive deterioration of vulnerable DA neurons in the SNpc may be attributed to cellular disturbances characterized by mitochondrial dysfunction, disruption of the autophagy–lysosome system, misfolding and aggregation of misfolded proteins, and endoplasmic reticulum (ER) stress and neuroinflammation.^[^ ^3^, ^5^ ^]^ Notably, nearly 50% of DA neuron loss in the SNpc occurs when movement deficiencies appear; however, there is still a lack of efficient means for mediation to attenuate or prevent DA neuron death.^[^ ^2^, ^3^ ^]^ Thus, mechanistic and therapeutic targets for rescuing DA neuron degeneration are strongly desirable.

DA neuron replenishment represents a cardinal target for both idiopathic and symptomatic PD therapeutics; however, as the disease progresses, long‐term therapy may induce dyskinesia and other motor complications.^[^ ^6^ ^]^ Although several reprogrammable and gene‐editing approaches have yielded promising benefits for alleviating neurological deficits and DA neuron loss in PD animal models, investigations are still preclinical, and technical challenges and ethical considerations must be overcome before their clinical application.^[^ ^7^ ^]^

Tyrosine hydroxylase (TH) catalyzes the formation of L‐3,4‐dihydroxyphenylalanine (l‐DOPA), the rate‐limiting step in the biosynthesis of DA, and TH is, therefore, recognized to be a characteristic hallmark of the DA neuron. Nigrostriatal TH protein has been widely reported to be lost in PD patients,^[^ ^8^, ^9^ ^]^ and its loss correlates with the progression of DA neuron death. Emerging evidence reveals that TH is ubiquitinated in PD, which results in its degradation by the ubiquitin–proteasome system.^[^ ^10^, ^11^, ^12^ ^]^ Importantly, pathological TH is a potential source and contributor to the generation of reactive oxygen species (ROS), which is a common feature of many neurodegenerative disorders, including PD.^[^ ^13^, ^14^ ^]^ Thus, TH may be a promising candidate target protein for developing new treatments of PD,^[^ ^15^ ^]^ though the potential of targeting TH ubiquitination has not been extensively researched.

Recently, 4,4′‐dimethoxychalcone (DMC) has been identified as a natural autophagy inducer with antiaging properties.^[^ ^16^ ^]^ Because DMC promotes cytoprotection and autophagy across species, we speculated that it may be neuroprotective in PD treatment. However, the blood brain barrier (BBB) would be likely to impede the potential intervention efficacy of DMC, and the proposed mechanisms of DMC for PD remain unexplored. Over the last decades, increasing efforts have focused on developing effective nanocarrier systems that cross the BBB to treat neurodegenerative diseases.^[^ ^17^, ^18^ ^]^ Appropriation of brain‐targeting ligands, such as transferrin receptors and glucose transporters, facilitates BBB penetration by nanodelivery systems for more effective neurodegenerative diseases therapy.^[^ ^19^, ^20^ ^]^ Although several systems have been established to delivery nanomedicines that target *α*‐synuclein, as another pathological hallmark in PD progression,^[^ ^20^, ^21^ ^]^ targeted delivery systems to treat PD by restoring DA activity remain unexplored.

**Scheme 1:**
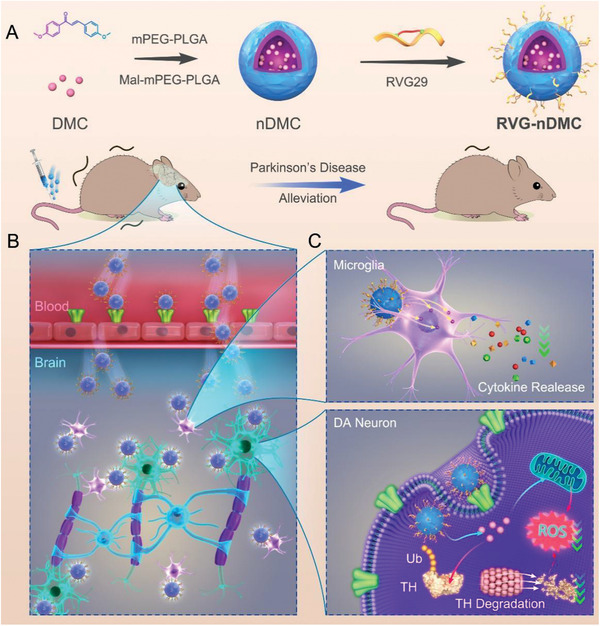
The preparation and proposed mechanism of RVG‐nDMC for PD intervention. A) Schematic illustration of the synthesis of RVG‐nDMC. RVG‐nDMC is composed of DMC modified with polymer nanoparticle linked with RVG peptide. B) Proposed mechanism by which RVG‐nDMC ameliorates motor deficits and nigral DA neuron death in the MPTP‐induced PD mouse model. C) Briefly, RVG‐nDMC penetrates the BBB and accumulates in DA neurons and microglia in the brain. Specifically, RVG‐nDMC reverses MPTP‐induced TH ubiquitination and degradation, as well as oxidative stress. Additionally, RVG‐nDMC exerts antiinflammatory effects in the PD mouse model.

In this study, a BBB‐penetrating peptide (RVG29)‐based “Trojan horse” system (designated as RVG‐nDMC) was prepared as a strategy for PD therapy by substantia nigra‐targeted delivery of DMC in a mouse model (**Scheme** **1**A). RVG‐nDMC penetrated the blood brain barrier and enhanced the brain‐targeted delivery efficiency by binding the nicotinic acetylcholine receptor (nAchR), which was subsequently internalized by DA neurons and microglia (Scheme 1B). Importantly, in vivo and in vitro studies demonstrated that RVG‐nDMC reduces TH ubiquitination and degradation independent of regulating TH phosphorylation and decreases subsequent ubiquitinated TH‐mediated oxidative stress in DA neurons. RVG‐nDMC also alleviates inflammatory cytokine release in microglia (Scheme 1C). Moreover, transcriptome sequencing and metabolomics analysis confirmed that RVG‐nDMC ameliorates motor deficits and nigral DA neuron death in PD mice without observable toxic effects in nontargeted organs, indicating great potential of RVG‐nDMC for clinical translation and neuroprotection in PD.

## 2 Results and Discussion

### 2.1 Preparation and Characterization of RVG‐nDMC

**Figure 1:**
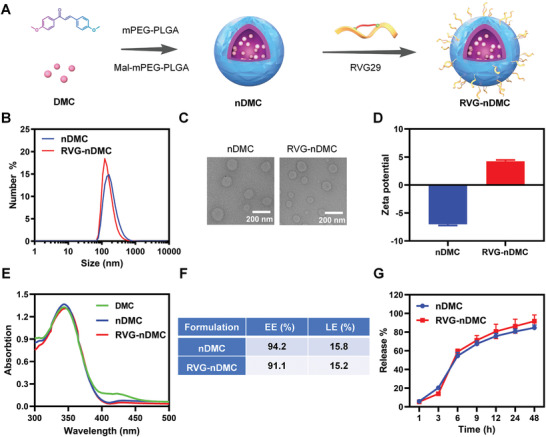
Characterization of nDMC and RVG‐nDMC. A) Schematic illustration of the synthesis process of RVG‐nDMC nanoparticles. Evaluation of nDMC and RVG‐nDMC by B) particle size analysis; C) TEM observation (Scale bars, 200 nm); and D) zeta potential analysis. *n* = 3 per group. E) UV–vis absorbance of DMC, nDMC, and RVG‐nDMC. F) EE and LE of DMC in nDMC and RVG‐nDMC were measured with a spectrophotometer. G) The drug release kinetics of nDMC and RVG‐nDMC. *n* = 3 per group. Results are expressed as the mean ± SEM. Statistical significance was determined by Student's *t*‐tests.

To obtain RVG‐nDMC, DMC was first physically encapsulated into the hydrophobic core of PEG‐b‐PLGA nanoparticles. PLGA20K‐b‐mPEG2K and PLGA20K‐PEG5K‐MAL were used to prepare nDMC, which was further modified with RVG29 peptide through a maleimide–thiol reaction (**Figure** **1**A). As shown in Figure 1B,C, the nDMC and RVG‐nDMC appeared as uniform nanosized and sub‐200 nm nanoparticles. Moreover, the positive charge of nDMC reversed to a negative charge after RVG29 peptide modification, which confirms that RVG29 peptide was successfully conjugated onto nDMC (Figure 1D). Similar characteristic absorption peaks of DMC, nDMC, and RVG‐nDMC were observed at 350 nm, demonstrating that DMC was efficiently loaded into nDMC and RVG‐nDMC (Figure 1E). The drug encapsulation efficiency (EE), loading efficiency (LE), and release behaviors of nDMC and RVG‐nDMC were measured by using a standard curve of the UV–vis absorbance of DMC at 350 nm (Figure S1, Supporting Information), and the resulting EEs for nDMC and RVG‐nDMC were 94.2% and 91.1%, with LEs for nDMC and RVG‐nDMC of 15.8% and 15.2%, respectively (Figure 1F). Furthermore, DMC underwent sustained released from nDMC and RVG‐nDMC under physiological conditions (Figure 1G), which has potential therapeutic advantages. Collectively, these results suggest successful preparation of RVG‐nDMC for assessment of the therapeutic benefit of DMC.

### 2.2 RVG‐nDMC Displays Widespread Cellular Distribution and Restores MPP^+^‐Damaged Cell Viability in MN9D Cells

**Figure 2:**
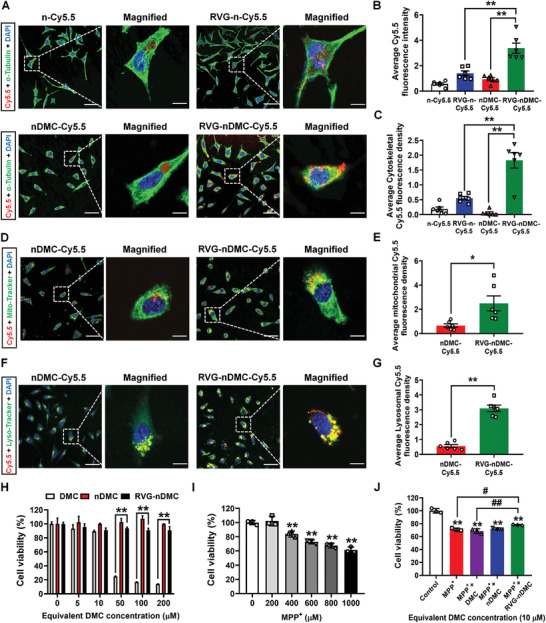
Biological effects of RVG‐nDMC on DA neuron‐derived MN9D cells. A) Representative images and B,C) quantitative fluorescence analysis of *α*‐Tubulin in MN9D cells after 6 h incubation with different Cy5.5‐labelled t nanoparticles. *n* = 6 per group. D) Representative images and E) quantitative fluorescence analysis of mitochondrial tracker (Mito‐tracker) in MN9D cells after 6 h incubation with Cy5.5‐labelled nDMC and RVG‐nDMC. *n* = 6 per group. F) Representative images and G) quantitative fluorescence analysis of lysosomal tracker (Lyso‐tracker) in MN9D cells after 6 h incubation with Cy5.5‐labelled nDMC and RVG‐nDMC. *n* = 6 per group. Scale bars, 50 µm for the original images and 10 µm for the magnified images. H) Cell viability of MN9D cells treated with increasing concentrations of DMC, nDMC, and RVG‐nDMC. *n* = 3 per group. I) Cell viability of MN9D cells treated with increasing concentrations of MPP^+^. *n* = 3 per group. J) Cell viability of MPP^+^ pretreated MN9D cells incubated with DMC, nDMC, and RVG‐nDMC. *n* = 3 per group. Results are expressed as the mean ± SEM. ^**^ *p* < 0.01. ^##^ *p* < 0.01, ^#^ *p* < 0.05 vs MPP^+^ + RVG‐nDMC group. The Unpaired Student's *t*‐test was used for comparison between two groups. One‐way ANOVA with a Tukey post‐hoc analysis was used for comparison among multiple groups.

RVG29 peptide has been shown to recognize neural nAchR or gamma‐aminobutyric acid (GABA) receptor, which may promote the intracellular delivery in nAchR or GABA‐overexpressed neural cells.^[^ ^22^ ^]^ Therefore, we investigated the cellular uptake behavior of RVG29‐nDMC in DA neuron‐derived MN9D cells. As shown in **Figure** **2**A, RVG29‐nDMC was efficiently internalized by MN9D cells. Intriguingly, RVG‐nDMC promoted more cytoskeletal accumulation than RVG‐n did, suggesting that DMC conjugation may promote nanoparticle entry (Figure 2A–C). To quantitatively analyze differences in the cellular uptake of nDMC and RVG‐nDMC, we employed flow cytometry. The cellular uptake of RVG‐nDMC was significantly increased as compared with the cellular uptake of nDMC (Figure S2A,B, Supporting Information), which is consistent with the microscopy results. These findings suggest that intracellularly released DMC may promote the membrane distribution or recycling of nAchR or GABA receptor, which may induce its sequential binding with RVG29‐nDMC and increase nanoparticle entry. Because DMC has been reported to be an autophagy inducer with antiaging properties,^[^ ^16^ ^]^ we examined the interactions of RVG‐nDMC with the mitochondria and lysosomes. RVG‐nDMC significantly accumulated in the mitochondria and lysosome as determined by costaining with mitochondrial tracker (Mito‐tracker), mitochondrial marker, pyruvate dehydrogenase E1*α* (PDH‐E1*α*), and lysosomal tracker (Lyso‐tracker) (Figure 2D–G; Figure S3A,B, Supporting Information). In a previous study, a modified RVG peptide (RVG9R) was used to deliver short interfering RNA (siRNA) to the brain, and RVG9R‐loaded siRNA was found to specifically interact with the Mitochondrial Complex I.^[^ ^23^ ^]^ Thus, although there are no mitochondria‐targeting ligands on the surface of the nanoparticles, it would be of interest in the future to determine whether RVG29 can physically interact with mitochondria.

Further analysis suggests that modification of DMC to nDMC and RVG‐nDMC significantly decreased the undesired cytotoxicity of DMC, especially at higher DMC concentrations (50 × 10^−6^, 100 × 10^−6^, and 200 × 10^−6^ m) (Figure 2H). We further constructed an in vitro PD model by using neurotoxin 1‐methyl‐4‐ phenylpyridinium (MPP^+^) to treat MN9D cells with an optimized concentration of MPP^+^ at 1000 × 10^−6^ m (Figure 2I). RVG‐nDMC, but not free DMC or nDMC, extensively restored MPP^+^‐decreased cell viability (Figure 2J). These results reveal that RVG‐nDMC targets mitochondria and lysosomes upon internalization, is relatively nontoxic, and shows beneficial effects in an in vitro model of PD.

### 2.3 Brain Targeting and Distribution of RVG‐nDMC in the SNpc

**Figure 3:**
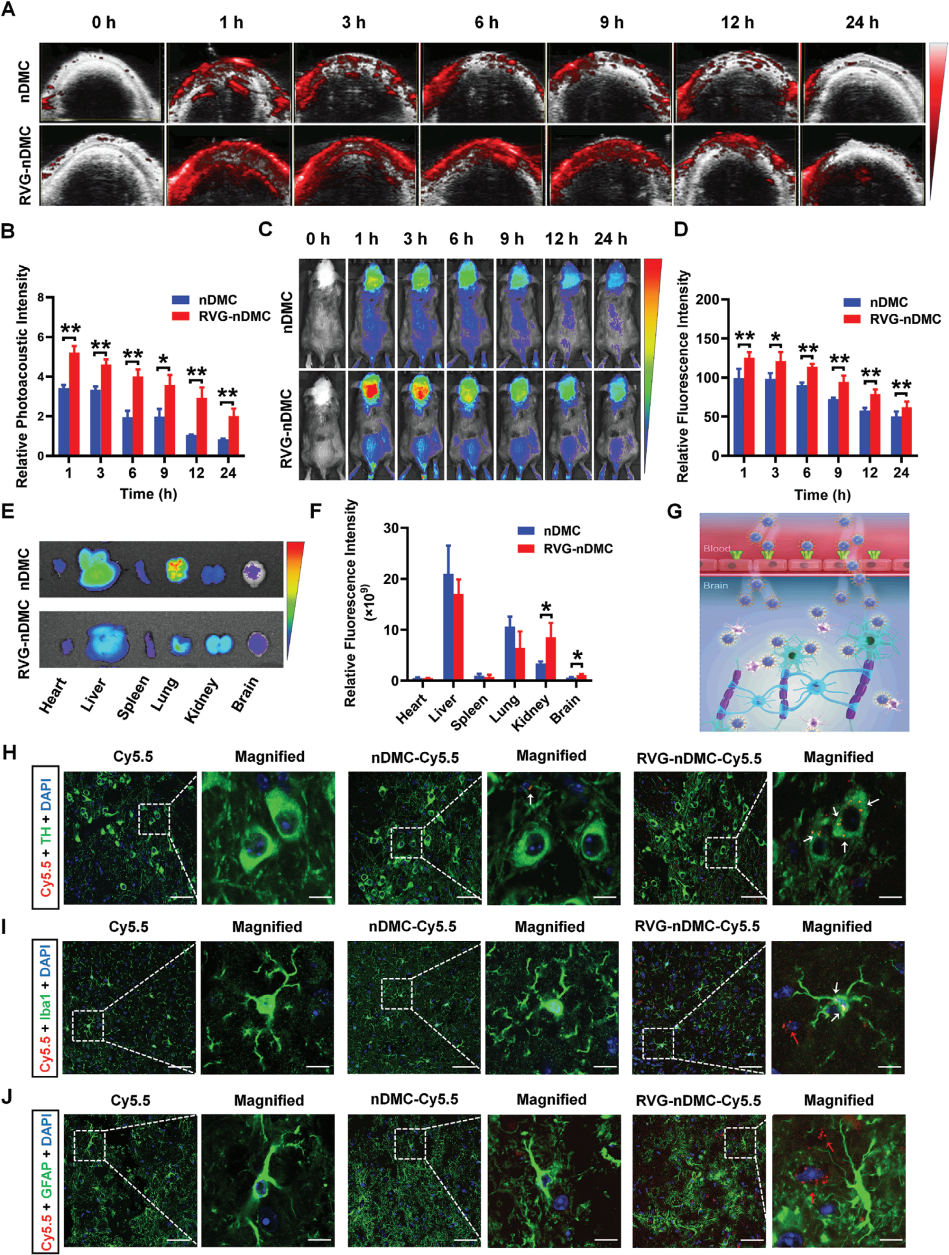
Brain Targeting Delivery of RVG‐nDMC. A) Real time photoacoustic imaging and B) corresponding photoacoustic signal analysis of mice after intravenously injection of nDMC or RVG‐nDMC. *n* = 3 per group. C) Real time fluorescence imaging and D) corresponding fluorescence analysis of mice after intravenously injection of Cy5.5‐labelled nDMC or RVG‐nDMC. *n* = 3 per group. E) Ex vivo imaging and F) corresponding fluorescence analysis of the sacrificed tissues after intravenously injection of Cy5.5‐labelled nDMC or RVG‐nDMC at 48 h. *n* = 3 per group. G) Schematic illustration of the proposed mechanism of RVG‐nDMC for brain targeting delivery. H–J) Representative images of TH, Iba1, and GFAP staining in SN derived from mice treated with Cy5.5, nDMC‐Cy5.5, or RVG‐nDMC‐Cy5.5 at 6 h postinjection. White arrows in the enlarged details of the right column show the presence of nanoparticles in DA neurons and microglia. Red arrows show the presence of nanoparticles outside of astrocytes and microglia. Scale bars, 50 µm for the original images and 10 µm for the magnified images. Results are expressed as the mean ± SEM. ^**^ *p* < 0.01, ^*^ *p* < 0.05. The Unpaired Student's *t*‐test was used for comparison between two groups.

The BBB impedes the efficiency of brain‐targeted drug delivery, which restricts the efficiency of PD interventions. Therefore, we determined whether RVG‐nDMC may have the ability to overcome this limitation. Indeed, our results indicate that RVG‐nDMC possesses favorable brain‐targeting ability. Compared to nDMC, RVG‐nDMC exhibited a greater efficiency in brain‐targeting delivery as evaluated by photoacoustic imaging (**Figure** **3**A,B), fluorescence imaging (Figure 3C,D), and ex vivo fluorescence imaging (Figure 3E,F). Notably, the distribution of RVG‐nDMC‐Cy5.5 in the brain was earlier and prolonged relative to the distribution of nDMC‐Cy5.5. These results suggest that RVG‐nDMC can penetrate the BBB with more extensive distribution than for nDMC, which is of potential advantage for PD intervention (Figure 3G).

The SNpc is a well‐known vulnerable brain region in PD.^[^ ^24^ ^]^ Thus, to further evaluate the SNpc targeting ability of RVG‐nDMC, we examined the distribution of RVG‐nDMC in the SNpc at different time points (1, 6, and 24 h) using differential neural cell markers (TH for DA neuron, ionized calcium binding adapter protein 1 (Iba1) for microglia, and glial fibrillary acidic protein (GFAP) for astrocytes) after intravenous injection. RVG‐nDMC was localized in DA neurons at 1, 6, and 24 h postinjection (Figure 3H; Figures S4A and S5A, Supporting Information), indicating favorable DA neuron targeting ability. Intriguingly, Cy5.5 signals were also observed in microglia at 1, 6, and 24 h postinjection (Figure 3I; Figures S4B and S5B, Supporting Information). Microglia express much less nAchR and GABA receptor than neurons and function as resident professional phagocytes in the brain.^[^ ^25^ ^]^ Therefore, we speculate that RVG‐nDMC might be engulfed by microglia in the SNpc, which play a clear role in the inflammation of PD. In contrast, no RVG‐nDMC was observed in astrocytes (Figure 3J; Figures S4C and S5C, Supporting Information). Thus, these results reveal that RVG‐nDMC penetrates the SNpc and localizes primarily in DA neurons, with additional localization in microglia.

### 2.4 RVG‐nDMC Administration Improves Motor Deficits in PD Mice

**Figure 4:**
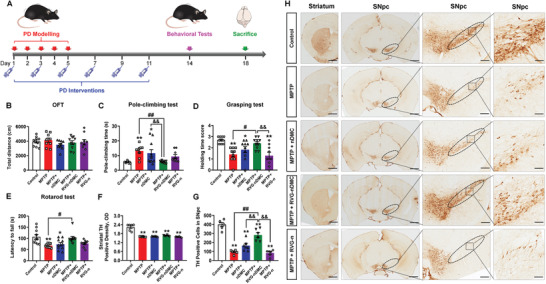
RVG‐nDMC attenuates motor deficits and DA neuron loss in PD mice. A) Experimental design for RVG‐nDMC administration in MPTP‐induced PD mice. B) Total distances travelled in the open‐field of PD mice treated with nDMC, RVG‐n, and RVG‐nDMC. C) The pole‐climbing test was used to examine the bradykinesia of PD mice. D) The grasping test was used to further examine the grip strength of PD mice. E) The rotarod test was used to examine the motor coordination of PD mice. *n* = 10, 9, 10, 10, and 9 for Control, MPTP, MPTP + nDMC, MPTP + RVG‐nDMC, and MPTP + RVG‐n groups, respectively. F,G) Quantification of the TH‐positive density in the striatum and TH‐positive neurons in the SN. *n* = 6 per group. H) Immunohistochemical staining of TH‐positive neurons in the striatum and SNpc in Control, MPTP, MPTP + nDMC, MPTP + RVG‐nDMC, and MPTP + RVG‐n groups. The ellipses in the middle column of panel H show the boundaries of the SNpc and the middle‐column boxes denote the areas that are expanded in the right‐hand columns. Scale bars, 1 mm for images in striatum; 800, 100, and 50 µm for the series of images in SNpc. Results are expressed as the mean ± SEM. ^**^ *p* < 0.01, ^*^ *p* < 0.05 vs Control. ^##^ *p* < 0.01, ^#^ *p* < 0.05 vs MPTP group. ^&&^ *p* < 0.01, ^&^ *p* < 0.05 vs MPTP + RVG‐nDMC group. Statistical significance was determined by one‐way ANOVA and Tukey tests for post‐hoc comparisons.

To evaluate whether RVG‐nDMC may provide in vivo PD intervention efficacy via DA neuron‐targeted delivery, we employed the 1‐methyl‐4‐phenyl‐1,2,3,6‐tetrahydropyridine (MPTP)‐induced PD mouse model.^[^ ^26^ ^]^ MPTP was administrated intraperitoneally continuously over 5 days to induce a subacute PD state (**Figure** **4**A). Simultaneously, nDMC, RVG‐nDMC (equivalent dose of DMC), or the control vehicle were administered intravenously every other day for 12 days. After 3 days, behavioral tests were carried out to evaluate the protective effects of RVG‐nDMC against motor dysfunction mediated by MPTP administration. No obvious significant differences were observed in the total travelled distance, time spent in the center, or movement speed in the open field test (Figure 4B; Figure S6A,B, Supporting Information). However, RVG‐nDMC reversed the MPTP‐induced increased pole‐climbing time and decreased the holding time and latency to fall in the rotarod test; and the improved behavior performance was not observed for nDMC or RVG‐n alone, suggesting that both the nanocarrier and DMC moieties of RVG‐nDMC are required for improved motor performance (Figure 4C–E).

**Figure 5:**
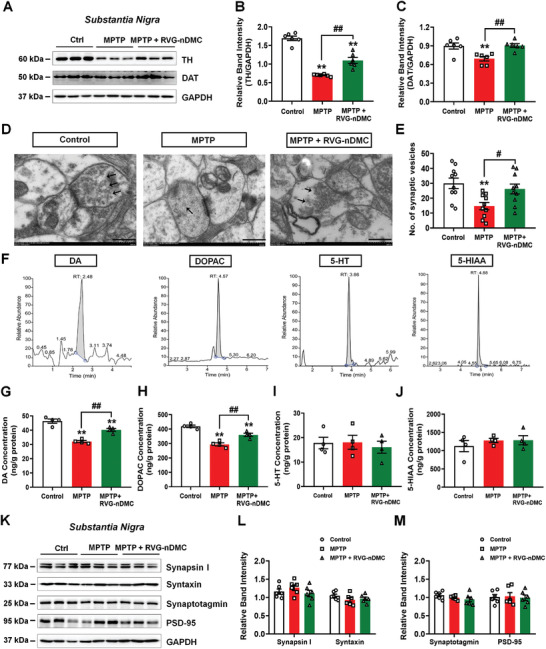
RVG‐nDMC increases TH expression and DA synthesis in PD mice. A–C) Representative blots and quantification showing TH and DAT expression levels in the SN of Control, MPTP, and MPTP + RVG‐nDMC groups. *n* = 6 per group. D) Ultrastructural analysis of synaptic vesicles in the SN of the three groups. Scale bars, 500 nm. Black arrows show the morphological synaptic vesicles. E) Quantification of synaptic vesicles in the SN of the three groups. *n* = 10 per group. F) Typical HPLC chromatograms of DA, DOPAC, 5‐HT, and 5‐HIAA. G–J) The levels of DA, DOPAC, 5‐HT, and 5‐HIAA were assessed by HPLC‐MS/MS analysis. *n* = 4 per group. K–M) Representative blots and quantification showing Synapsin, Syntaxin, Synaptotagmin, and PSD‐95 expression levels in the SN of the three groups. *n* = 6 per group. Results are expressed as the mean ± SEM. ^**^ *p* < 0.01 vs Control. ^##^ *p* < 0.01, ^#^ *p* < 0.05 vs MPTP group. Statistical significance was determined by one‐way ANOVA and Tukey tests for post‐hoc comparisons.

TH is the rate‐limiting enzyme in DA biosynthesis, and its dysfunction may underlie DA neuron loss in PD.^[^ ^4^, ^8^, ^27^ ^]^ Therefore, we evaluated the effect of RVG‐nDMC on TH levels after MPTP treatment. No obvious differences were observed in the striatal TH density between the different treatment groups (Figure 4F,H). However, the MPTP + RVG‐nDMC group had greater amounts of TH‐positive neurons in the SNpc as compared with the MPTP, MPTP + nDMC, and MPTP + RVG‐n groups (Figure 4G,H). Furthermore, RVG‐nDMC was verified to increase nigral TH and dopamine transporter (DAT) expression in PD mice by Western blot analysis (**Figure** **5**A–C). Consistently, RVG‐nDMC increased the number of DA synaptic vesicles in the substantia nigra (SN) in the PD model mice as evaluated by transmission electron microscopy (TEM) of the ultrastructural morphology of synaptic vesicles (Figure 5D,E). These results suggested that RVG‐nDMC improves MPTP‐damaged DA synaptic plasticity. Notably, based on high‐performance liquid chromatography coupled with tandem mass spectrometry (HPLC/MS‐MS), RVG‐nDMC also reversed the MPTP‐induced decrease in DA, as well as 3,4‐dihydroxyphenylacetic acid (DOPAC), a product of the metabolism of DA (Figure 5F–H). In contrast, RVG‐nDMC showed no obvious effect on the levels of other neurotransmitters, such as serotonin (5‐hydroxytryptamine, 5‐HT) and 5‐hydroxyindoleacetic acid (5‐HIAA), the chief end‐product of serotonin metabolism (Figure 5F,I,J). Moreover, there was no obvious effect on the expression of several synaptic proteins after treatment with MPTP and RVG‐nDMC, such as Synapsin, Syntaxin, Synaptotagmin, and PSD‐95 in the SN (Figure 5K–M), suggesting RVG‐nDMC may not alter nigral synaptic transmission in the PD model mice. Therefore, these results are consistent with specificity in the effect of RVG‐nDMC in ameliorating DA loss in the SNpc.

Given that RVG‐nDMC becomes localized, in part, in the liver, lung and kidney of mice after intravenous administration, we examined potential adverse effects of RVG‐nDMC on non‐targeted organs. After RVG‐nDMC administration, no detectable pathological changes were observed in the liver, heart, kidney, lung or spleen from normal mice as evaluated by H&E staining (Figure S7, Supporting Information). Consistently, no detectable alterations were observed in serum biochemical indicators of liver or kidney function following nDMC and RVG‐nDMC administration (Table S1, Supporting Information). However, nDMC resulted in observable renal tubular atrophy and interstitial loosening in the kidney without changes in serum biochemical indicators of kidney function (black arrows in Figure S7 and Table S1 in the Supporting Information). Substantially more RVG‐nDMC as compared with nDMC could be observed in the kidneys of the mice (Figure 3E,F). This may be partially due to the expression of nAchR in the kidney,^[^ ^28^ ^]^ which facilitates the binding of RVG29 peptide. The slight kidney damage upon nDMC treatment observed in this study may result from the interplay between MPTP and dysfunctional uptake of nDMC in the kidney. Moreover, the surface charge of nanoparticles dictates the suborgan distribution of nanoparticles in the kidney, which also may affect the metabolism of nanoparticles.^[^ ^29^ ^]^ As illustrated in Figure 1D, the modification of RVG29 peptide reverses the negatively charged nDMC to positively charged RVG‐nDMC, which might further affect drug metabolism and drug effects. Importantly, we did not find significant changes in biochemical indicators of kidney function. Thus, we hypothesize that this may be a temporary structural dysfunction, and further study is needed to evaluate the safety and toxicity of DMC in the future. However, these results indicate overall that RVG‐nDMC can efficiently exert neuroprotection without causing obvious side effects.

### 2.5 RVG‐nDMC Administration Inhibits TH Ubiquitination in PD Mice

**Figure 6:**
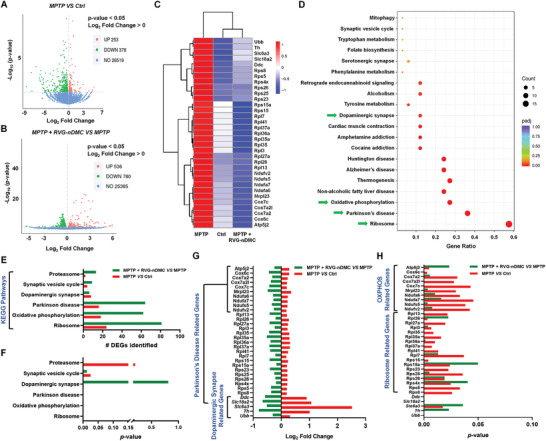
RNA‐sequencing analysis of RVG‐nDMC treatment in the MPTP‐induced PD mouse model. A,B) Volcano plots showing the DEGs between the MPTP and Control groups, as well as the MPTP + RVG‐nDMC and MPTP groups. C) Hierarchical clustering of 33 representative MPTP + RVG‐nDMC‐reversed DEGs that were increased in MPTP as compared with Control group. D) KEGG pathways enriched by DEGs among these three groups. Green arrows show the PD‐related pathways. E,F) The number and *p* value of identified DEGs in PD‐related KEGG pathways. G,H) The Log_2_ fold change and *p* value of 33 individual DEGs enriched in the KEGG pathways in (E) and (F).

Next, we investigated the proposed mechanism of RVG‐nDMC for restoring TH positive neurons by RNA‐sequencing analysis. Volcano plots (**Figure** **6**A,B) and hierarchical clustering (Figure 6C) identified 33 representative differentially expressed genes (DEGs) that were increased by MPTP and also were reversed by MPTP + RVG‐nDMC treatment. For further insight into the potential pathways involved in these DEGs, we performed Kyoto Encyclopedia of Genes and Genomes (KEGG) analysis (Figure 6D; Figure S8, Supporting Information). Intriguingly, the top 3 DEG‐enriched pathways were “Ribosome,” “Parkinson's disease,” and “Oxidative phosphorylation,” while there also was high enrichment for the “Dopaminergic synapse” pathway (see green arrows in Figure 6D). For further verification, we examined the number and *p* value of identified DEGs for the PD‐related KEGG pathways, including “Ribosome,” “Parkinson's disease,” “Oxidative phosphorylation,” “Dopaminergic synapse,” “Synaptic vesicle cycle,” and “Proteasome” (Figure 6E,F). Additionally, the Log_2_ fold change and *p* value of the 33 individual DEGs enriched in these pathways were measured. The results demonstrate that the overlapping dopaminergic synapse and PD‐related genes (*Th*, *Ddc*, *Slc18a2*, *Slc6a3*, and *Ubb*) were increased in the SN of PD mice as compared with control mice, and that RVG‐nDMC decreased the mRNA expression of these genes, with similar results for 18 ribosome related genes (Figure 6G,H). Notably, the expression of specific genes encoding ribosomal proteins has been demonstrated to be decreased in the SN in the late stage of PD patients, with associated progressive neuronal loss in the SN.^[^ ^30^ ^]^ Therefore, the increased expression of ribosomal protein subunit genes might be compensatory to the DA neuron loss upon subacute MPTP damaged in SN. Consistently, further bioinformatic analysis verified that the altered genes have key roles in Ribosome, Parkinson's disease, Oxidative phosphorylation and Dopaminergic synapse pathways (Figure S9A–D, Supporting Information).

**Figure 7:**
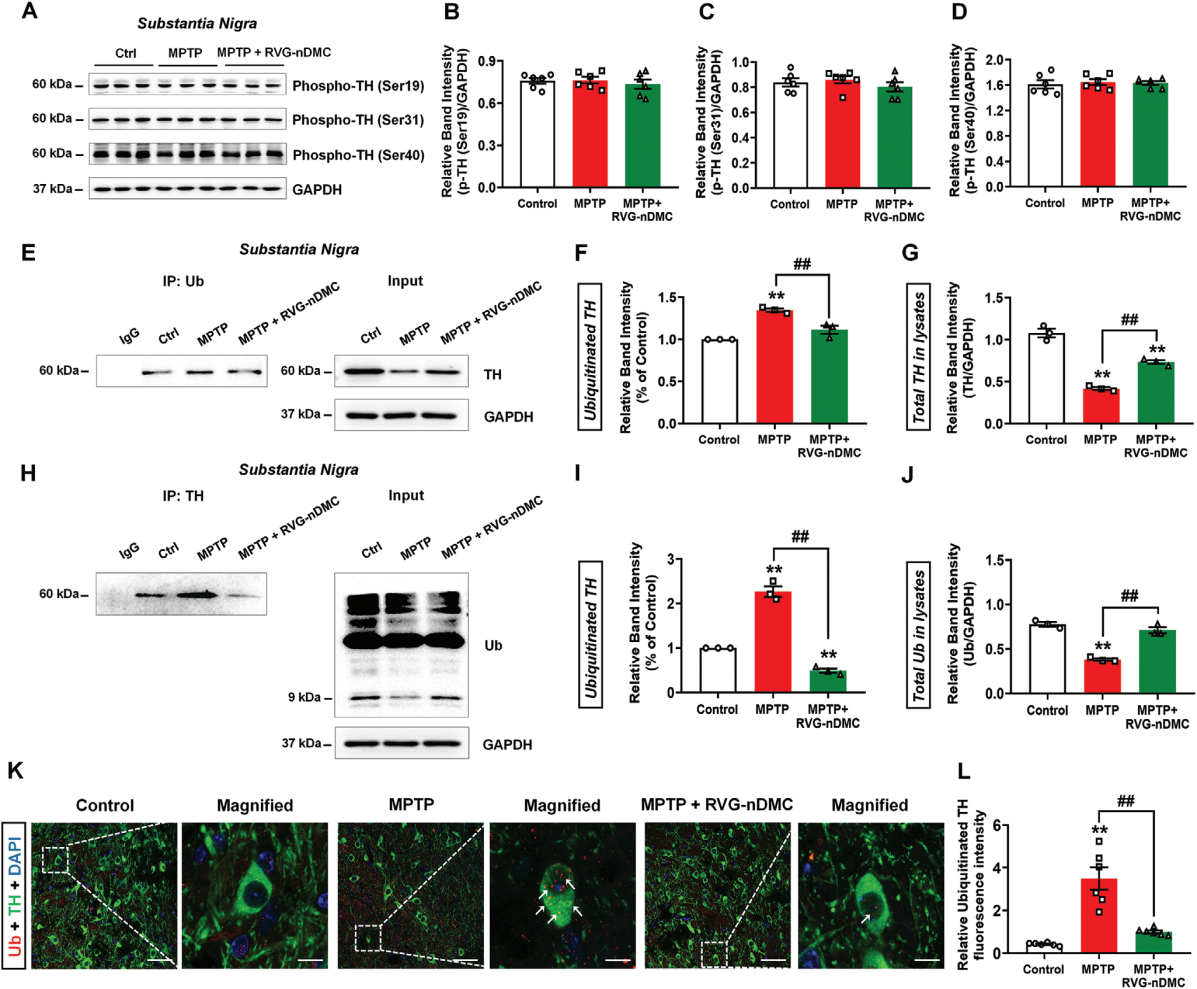
RVG‐nDMC reverses TH ubiquitination in the MPTP‐induced PD mouse model. A–D) Representative blots and quantification showing the expression levels of phosphorylated TH at Ser19, Ser31, and Ser40 in the SN of Control, MPTP, and MPTP + RVG‐nDMC groups. *n* = 6 per group. E–G) Representative blots and quantification of co‐IP assays showing the interaction between TH and Ub by using Ub as the capture antibody in the SN of the three groups. *n* = 3 per group. H–J) Representative blots and quantification of co‐IP assays showing the interaction between TH and Ub by using TH as the capture antibody in the SN of the three groups. *n* = 3 per group. K,L) Immunofluorescent staining and quantification of Ub within TH positive neurons. *n* = 6 per group. White arrows show the immunostaining of Ub in TH‐positive neurons. Scale bars, 50 and 10 µm for the original and magnification of images in (K). Results are expressed as the mean ± SEM. ^**^ *p* < 0.01 vs Control. ^##^ *p* < 0.01 vs MPTP group. Statistical significance was determined by one‐way ANOVA and Tukey tests for post‐hoc comparisons.

Because we found the opposite expression pattern for TH mRNA and protein upon RVG‐nDMC treatment in the MPTP mouse model, we further explored the related mechanisms. Mechanistically, the activity of TH is subject to long‐term regulation of gene expression (enzyme stability, transcriptional and translational regulation, and RNA stability), as well as short‐term regulation of enzyme activity (allosteric regulation, feedback inhibition, and phosphorylation).^[^ ^31^ ^]^ Previous work has demonstrated that TH protein is ubiquitinated and degraded by the ubiquitin–proteasome system, dependent, in part, on its phosphorylation, and that this ubiquitination is linked DA neuron loss.^[^ ^11^, ^12^, ^32^ ^]^ Therefore, we sought to evaluate the effects of RVG‐nDMC on TH protein phosphorylation and ubiquitination. First, we evaluated three phosphorylation sites on TH located in the N‐terminal regulatory domain.^[^ ^33^ ^]^ RVG‐nDMC showed no obvious effects on TH phosphorylation at Ser19, Ser31, and Ser40 (**Figure** **7**A–D). Next, we performed co‐immunoprecipitation (co‐IP) assays to determine whether TH is ubiquitinated upon MPTP treatment. A direct interaction between ubiquitin and TH was observed using capture antibody for either ubiquitin or TH, and RVG‐nDMC reduced this interaction, suggesting that RVG‐nDMC inhibits ubiquitination of TH (Figure 7E–J). Similar results were also observed by costaining of TH and ubiquitin in the SNpc (Figure 7K,L). Because RVG‐nDMC reverses the decreased TH expression induced by MPTP, these results are consistent with the possibility that RVG‐nDMC inhibits the degradation of ubiquitinated TH to restore its expression in the SN of PD mice. Generally, ubiquitination of TH appears to be triggered by enzyme phosphorylation; however, other factors also induce TH degradation via proteasomes independent of its phosphorylation. For example, the inflammatory cytokine, ciliary neurotrophic factor, leads to the ubiquitination of TH in a gp130‐dependent manner, and gp130 cytokines induce proteasomal degradation of TH through an extracellular signal‐regulated kinases1/2 (ERK1/2)‐dependent pathway.^[^ ^12^ ^]^ ERK5 signaling is suggested to regulate ubiquitination of TH in DAergic PC12 cells independent of its phosphorylation.^[^ ^34^ ^]^ Additionally, the MgATP‐ubiquitin–proteasome‐dependent pathway is also indicated to regulate TH ubiquitination, suggesting that energy metabolism may play a specific role in the degradation of TH.^[^ ^35^ ^]^ Given that MPTP induces energy depletion by damaging mitochondrial function,^[^ ^36^ ^]^ we hypothesize that abnormal energy metabolism mediated by MPTP administration may contribute to the ubiquitination of TH. Thus, these results suggest that potential signaling pathways may modulate the ubiquitination of TH independent of its phosphorylation.

### 2.6 RVG‐nDMC Administration Reduces Oxidative Stress

**Figure 8:**
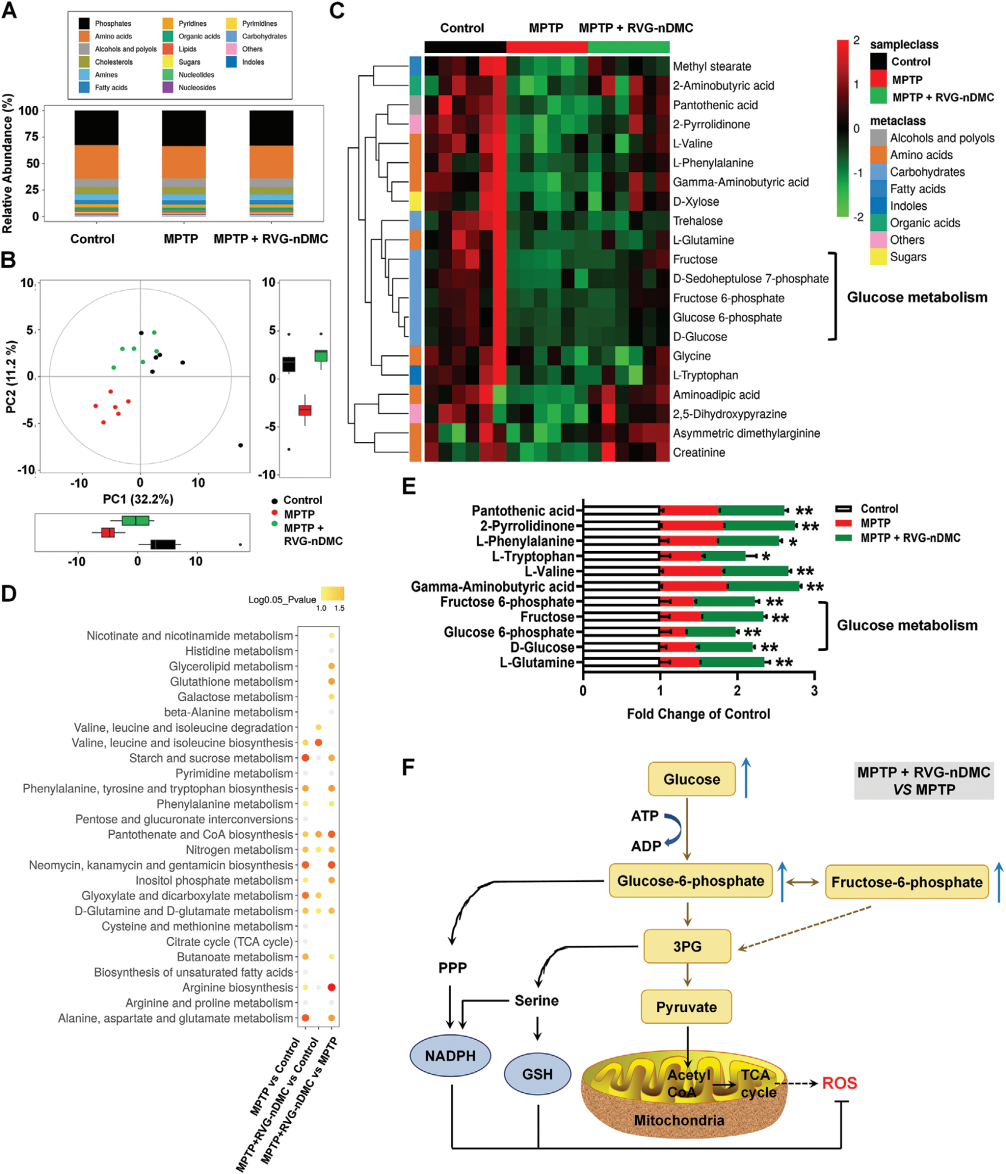
Metabolomic analysis of RVG‐nDMC treatment in the SN of PD mice. A) The relative abundances of representative metabolites in each group. B) Overall metabolic profiles of samples from the Control, MPTP, and MPTP + RVG‐nDMC groups using PCA plots. C) Z‐score plots showing the relative variations of each individual metabolite across all groups in the form of a heatmap. D) MPEA of potential pathways involved in the differential metabolite content between the indicated groups. E) Representative metabolites among the three groups. *n* = 6 per group. F) Schematic diagram showing RVG‐nDMC‐changed metabolites in the glucose metabolism pathway that affect oxidative stress in PD mice. Results are expressed as the mean ± SEM. ^**^ *p* < 0.01. Statistical significance was determined by one‐way ANOVA and Tukey tests for post‐hoc comparisons.

Pathological TH is a potential source and contributor of ROS generation, and ROS has been widely reported to induce DA neuron death.^[^ ^13^ ^]^ Therefore, we evaluated the potential effects of RVG‐nDMC on nigral metabolism in PD mice by performing GCTOF/MS (**Figure** **8**A; Figure S10A,B, Supporting Information). As shown in Figure 8B, a distinct separation of metabolites among the three groups of mice was observed in 2D boxplots and PCA score plots, indicating significant differences in the metabolic profiles. Z‐score plots showed relative variations of each individual metabolite across all groups, which is displayed in the form of a heatmap (Figure 8C). Next, metabolic pathway enrichment analysis (MPEA) was carried out to reveal potential pathways involved in the differential metabolites among these three groups (Figure 8D). Our results demonstrate that RVG‐nDMC improves glutathione metabolism by increasing Pyroglutamic acid and Ornithine (Figure S11A–C, Supporting Information). The polyamines putrescine, spermidine, and spermine are derived from ornithine under the catalyzation of ornithine decarboxylase.^[^ ^37^ ^]^ Because spermidine and spermine experimentally enhance longevity via autophagy induction,^[^ ^38^ ^]^ ample evidence supports their neuroprotective effects in PD. For example, spermidine protects PD from 6‐OHDA toxicity through its antioxidant and antiinflammatory properties, and it alleviates *α*‐synuclein neurotoxicity by inducing autophagy.^[^ ^39^ ^]^ It would therefore be of interest in the future to examine whether DMC exerts neuroprotective effects through the polyamine pathway.

RVG‐nDMC also reversed MPTP‐decreased d‐Glucose, Fructose, Glucose 6‐phosphate, and Fructose 6‐phosphate, which are enriched in glucose metabolism (Figure 8C,E). Previous studies have revealed an antioxidant‐like role of glucose, while glucose uptake inhibition has been shown to induce ROS accumulation.^[^ ^40^ ^]^ Thus, glucose metabolism may play an important role in regulating the balance of ROS production and scavenging in response to RVG‐nDMC (Figure 8F). On the one hand, glucose oxidation, through the pentose phosphate pathway (PPP), produces reducing equivalents in the form of nicotinamide adenine dinucleotide phosphate (NADPH), which are necessary to recycle antioxidant glutathione from its oxidized status.^[^ ^41^ ^]^ On the other hand, neuronal serine derived from glucose 6‐phosphate/3‐phosphoglycerate (3PG) metabolism is the substrate for NADPH and glutathione (GSH) synthesis.^[^ ^42^ ^]^

**Figure 9:**
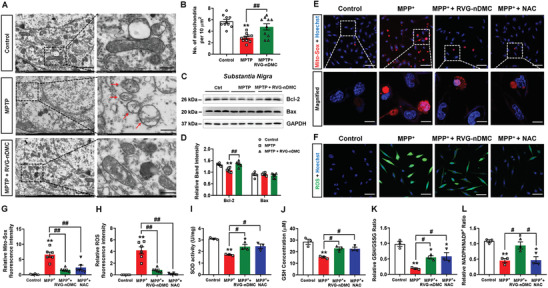
RVG‐nDMC attenuates oxidative stress in in vivo and in vitro PD models. A) Ultrastructural analysis of mitochondria in the SN of Control, MPTP, and MPTP + RVG‐nDMC groups. Red arrows indicate damaged mitochondria in the MPTP group. Scale bars, left, 2 µm; right, 500 nm. B) Quantification of the number of mitochondria in the three groups. *n* = 10 per group. C,D) Representative blots and quantification showing the Bcl‐2 and Bax expression levels in the SN for the three groups. *n* = 6 per group. E) Mito‐Sox was used to stain live cells upon RVG‐nDMC and NAC treatment in control or MPP^+^‐treated cells. Scale bars, upper, 50 µm; lower, 10 µm. F) Representative graphs of ROS generation upon RVG‐nDMC or NAC treatment in control or MPP^+^‐treated cells. Scale bars, 50 µm. G,H) Quantification of the relative Mito‐Sox and ROS fluorescence intensity. *n* = 6 per group. I,J) The content of SOD and GSH upon RVG‐nDMC or NAC treatment in control or MPP^+^‐treated cells. *n* = 3 per group. K,L) Relative GSH/GSSG and NADPH/NADP^+^ ratio after RVG‐nDMC or NAC treatment in control or MPP^+^‐treated cells. *n* = 3 per group. Results are expressed as the mean ± SEM. ^**^ *p* < 0.01 vs Control. ^##^ *p* < 0.01, ^#^ *p* < 0.05 vs MPTP or MPP^+^ group. Statistical significance was determined by one‐way ANOVA and Tukey tests for post‐hoc comparisons.

Pyruvate derived from glucose metabolism mitigates oxidative stress, including ROS generation, by sustaining the citric acid cycle (CAC, also called TCA) cycle.^[^ ^43^ ^]^ Therefore, we postulated that RVG‐nDMC may cause changes in mitochondrial metabolism. TEM results suggest that RVG‐nDMC reversed the MPTP‐decreased number of mitochondria and ameliorated mitochondrial morphological changes caused by MPTP, such as mitochondrial swelling and mitochondrial cristae disappearance (**Figure** **9**A, red arrows; quantified in Figure 9B). Furthermore, RVG‐nDMC restored the MPTP‐decreased expression of the mitochondrial‐associated antiapoptotic protein Bcl‐2, though no obvious effects on the pro‐apoptotic protein Bax were observed (Figure 9C,D). As further validation, we evaluated antioxidant‐like effects of RVG‐nDMC in MPP^+^‐treated DA‐derived MN9D cells. Mito‐Sox appeared oxidized in MPP^+^ treated cells, while RVG‐nDMC and *N*‐acetyl‐l‐cysteine (NAC) treatment significantly decreased oxidized Mito‐Sox and improved the mitochondrial damage (Figure 9E,G). Consequently, RVG‐nDMC and NAC reversed the MPP^+^‐induced ROS generation (Figure 9F,H). Notably, RVG‐nDMC and NAC also increased the superoxide dismutase (SOD) activity, GSH concentration and GSH/glutathione disulfide (GSSG) ratio (Figure 9I–K). Additionally, RVG‐nDMC, but not NAC, restored the reduced state of NADPH and its oxidized state (NADP^+^) ratio (Figure 9L). These results suggest that RVG‐nDMC may prevent DA loss by reducing oxidative stress.

Previously, DMC was revealed to exert neuroprotection by inducing autophagy in yeast and mice.^[^ ^16^ ^]^ Consequently, we evaluated the effects of RVG‐nDMC on autophagic indicators in PD mice. RVG‐nDMC slightly increased the number of autophagosomes in the SN of PD mice, though the differences were not statistically significant (Figure S12A,B, Supporting Information). Furthermore, the RVG‐nDMC group had increased LC3 II/I expression as compared with the MPTP group (Figure S12C,D, Supporting Information), suggesting that RVG‐nDMC might also induce autophagy in the subacute PD model mice, though the link between the neuroprotection of RVG‐nDMC in PD model mice and its regulation of autophagy is unclear.

### 2.7 RVG‐nDMC Administration Downregulates the Inflammatory Reaction

Because RVG‐nDMC can also be internalized by microglia (Figure 3I; Figures S4B and 5B, Supporting Information); and microglial‐mediated neuroinflammation underlies the pathogenesis of PD,^[^ ^44^ ^]^ we further examined the effects of RVG‐nDMC on the inflammatory cytokine expression profile. nDMC and RVG‐nDMC decreased mRNA levels of pro‐inflammatory cytokines, including IL‐1*β* and TNF‐*α* in the SN, and TNF‐*α* in the striatum of PD mice (Figure S13A,B,E, Supporting Information), suggesting general antiinflammatory properties of DMC. However, RVG‐nDMC showed no obvious effects on IL‐1*β* mRNA levels in the striatum or IL‐6 mRNA expression in the SN and striatum of PD mice (Figure S13C,D,F, Supporting Information). Because these cytokines are secreted proteins, we also examine their protein levels by ELISA. RVG‐nDMC significantly decreased the protein levels of IL‐1*β*, TNF‐*α*, and IL‐6 in the SN, as well as IL‐1*β* and TNF‐*α* in the striatum of PD mice (Figure S14A–E, Supporting Information), which is consistent with the mRNA results. Moreover, in multianalyte suspension arrays, RVG‐nDMC increased the expression of antiinflammatory cytokines, including IL‐4 and IL‐10, in the serum of PD mice (Figure S15C,G, Supporting Information). These results are consistent with an additional role for RVG‐nDMC in protecting against PD by downregulating the inflammatory reaction.

## 3 Conclusion

In this study, an RVG‐nDMC “Trojan horse” delivery system was constructed for brain‐targeted delivery of the antiaging drug DMC for PD intervention. RVG‐nDMC penetrated the BBB for enhanced delivery of DMC to the DA neurons and microglia in the SNpc in PD mice and ameliorated motor deficits and nigral DA neuron death without exhibiting adverse effects in the major organs after systemic administration. Moreover, RVG‐nDMC reversed TH ubiquitination and degradation, as well as oxidative stress, and exerted antiinflammatory effects in PD mice. Taken together, these findings identify RVG‐nDMC as a potentially powerful and clinically feasible approach for parkinsonian neuroprotection.

## 4 Experimental Section

##### Materials

DMC was purchased from Sigma‐Aldrich (St. Louis, MO, USA). Poly(ethylene glycol) methyl ether‐block‐poly(l‐lactide‐*co*‐glycolide) (PLGA20K‐b‐mPEG2K) and PLGA20K‐PEG5K‐MAL were purchased from Xi'an ruixi Biological Technology Co. Ltd. (Xi'an, China). Cy5.5 was purchased from Beijing Solarbio science & technology co. Ltd. (Beijing, China). MPP^+^ and MPTP were purchased from Sigma‐Aldrich (St. Louis, MO, USA). Anti‐TH (F‐11, sc‐25269), dopamine transporter (DAT, sc‐32258), and PDH‐E1*α* (sc‐377092) antibodies were purchased from Santa Cruz Biotechnology (Dallas, TX, USA). Anti‐synapsin І (#5297), Iba1(#17198), synaptotagmin (#14558), syntaxin (#18572), PSD‐95 (#3450), and Bax (#14796) antibodies were purchased from Cell Signaling Technology (Danvers, MA, USA). Bcl‐2 (16396‐1‐AP), *α*‐Tubulin (11224‐1‐AP), and GAPDH (60004‐1) antibodies were purchased from Proteintech Group (Rosemont, IL, USA). Antimicrotubule‐associated protein‐1 light chain 3 (LC3) II/І (ab48394) and p62 (ab5416) antibodies were purchased from Abcam (Cambridge, MA, USA). TH (Phospho‐Ser19) (E1A0037A), TH (Phospho‐Ser31) (E1A0038A), and TH (Phospho‐Ser40) (E1A0039A) antibodies were purchased from Enogene (Nanjing, China). DyLight 488 goat antimouse IgG (H+L) (70‐GAM4882) and DyLight 594 goat antirabbit IgG (H+L) (70‐GAR5942) antibodies were purchased from Multi Sciences (Hangzhou, China). Horseradish peroxidase (HRP)‐labeled goat antirabbit IgG, HRP‐labeled goat antimouse IgG and Mito‐tracker were purchased from Beyotime Biotechnology (Shanghai, China). MitoSOX Red Mitochondrial Superoxide Indicator was purchased from Thermo Fisher Scientific (Waltham, MA, USA).

##### Preparation of RVG‐nDMC

A mixture of DMC (4 mg), PLGA20K‐b‐mPEG2K (18 mg), and PLGA20K‐PEG5K‐MAL (2 mg) in CHCl_3_ (1 mL) was added to ethanol solution (4%) under sonication for 5 min. The ratio of PLGA20K‐b‐mPEG2K and PLGA20K‐b‐PEG5K‐MAL was optimized to balance the stability with the targeting ability of RVG‐nDMC, which was also done in a previous report.^[^ ^17^ ^]^ nDMC was obtained by evaporating the solution at 30 °C to remove the CHCl_3_. To prepare RVG‐nDMC, YC‐30 peptide was conjugated to nDMC via a maleimide–thiol reaction. Briefly, nDMC was mixed with an excess amount of YC‐30 peptide and incubated in PBS for 12 h at room temperature (RT). Subsequently, the solution was dialyzed against distilled water to remove the unconjugated YC‐30.

##### Characterizations of nDMC and RVG‐nDMC

The size distribution and *ζ*‐potential of nDMC and RVG‐nDMC were measured on a Zetasizer Nano ZS (Malvern, U.K.). The morphology of nDMC and RVG‐nDMC was observed by transmission electron microscopy (JEM‐1400PLUS, Japan). The absorption spectra were obtained by UV/vis spectrometry (Lambda 35, Perkin‐Elmer, USA). The fluorescence spectra were analyzed by fluorescence spectroscopy (LS55 luminescence spectrometer (Perkin‐Elmer).

##### Drug Release Analysis

The drug release kinetics of nDMC and RVG‐nDMC were measured using a dialysis method under physiological conditions. In brief, 1 mL nDMC and RVG‐nDMC (containing 2 mg mL^−1^ DMC) were suspended in PBS (1 mL, pH 7.4) in a dialysis bag (molecular weight cutoff, 100 kDa). Then, the solutions were dialyzed against PBS (30 mL, pH 7.4) at 37 °C. At different time points, the absorbance of the DMC was measured by UV–vis spectrometry, and the drug release kinetics were calculated by using a standard curve of DMC in PBS with the absorption at 350 nm.

##### Drug Loading and Encapsulation Efficiency

Freeze‐dried nDMC and RVG‐nDMC were dissolved in CHCl_3_. Subsequently, the solution was rotary evaporated to remove CHCl_3_. The DMC was dissolved in distilled water and measured by UV/vis spectrometry. The loading efficiency and encapsulation efficiency were calculated as follows:

LE = weight of DMC in nDMC or RVG‐nDMC/weight of nDMC or RVG‐nDMC × 100%

EE = weight of DMC in nDMC or RVG‐nDMC/weight of total DMC × 100%

##### Animals

Eight‐week‐old C57BL/6 mice, weight about (24 ± 2) g, were purchased from SPF Biotechnology Co., Ltd. (Beijing, China). The mice were housed in the experimental animal center of Guangzhou Medical University with a 12 h dark/light cycle at ambient temperature (22 ± 1) °C and relative humidity (60 ± 5)%. The experimental methods used were in compliance with the Institutional Animal Care and Use Committee of Guangzhou Medical University (Approval number: GY2020‐041) and National Institute of Health guidelines on the care and use of animals (NIH Publications No. 8023, revised 1978).

##### PD Mouse Model Induction and Drug Treatment

The MPTP‐induced PD mouse model was established according to the previous reports.^[^ ^45^ ^]^ MPTP was administrated intraperitoneally continuously over 5 days to induce the subacute PD model. Simultaneously, nDMC, RVG‐nDMC (equivalent dose of DMC), and unloaded RVG vehicle were administered intravenously starting on the day of the first MPTP injection once every other day for 12 days. After 3 days, behavioral tests were carried out.

##### Assessment of Brain Targeting Delivery

Mice were intravenously injected with nDMC or RVG‐nDMC (200 µL, containing 3 mg mL^−1^ DMC and 20 µg mL^−1^ Cy5.5). Afterward, the real time fluorescence was quantitatively analyzed by using the IVIS imaging system (Excitation wavelength: 674 nm, Emission wavelength: 692 nm). After 48 h, the major organs (heart, liver, spleen, lung, kidneys, brain) were sacrificed and analyzed by using the IVIS imaging system. Simultaneously, photoacoustic (PA) images of Cy5.5 were obtained and quantitatively analyzed on a preclinical photoacoustic computerized tomography scanner (Vevo LAZR‐X, Canada) at specified time intervals. Cy5.5 was used to label RVG‐nDMC for in vitro and in vivo pharmacokinetics analysis.

##### Open Field Test

Mice were placed in the bottom center of a box and filmed by EthoVisione XT software (Beijing, China) for 15 min. The movement speed, total travelled distance, and time spent in the central area were recorded.

##### Rotarod Test

Mice were trained on the Rotarod (Ugo basile SRL, Gemonio, VA, Italy) at a speed of 10 rpm for 3 days. During the test, the speed of the Rotarod accelerated from 4 rpm uniformly to 40 rpm within 5 min, and the time to falling was recorded.

##### Pole‐Climbing Test

Mice were placed head up on the top of a pole with a length of 75 cm and diameter of 9 mm. The total time that it took the mice to climb to the ground was recorded.

##### Grasping Test

Two front paws of mice were suspended on a horizontal wire (diameter l mm, 30 cm from the ground) for 10 s, and their abilities to grasp a wire with their hind legs was measured. The grasping score was as follows: The mouse grasped the wire with both hind legs, 3; the mouse grasped the wire with only one hind limb, 2; the mouse could not grasp the wires with either hind limb, 1; the mouse dropped, 0. Results were averaged from three measurements.

##### MN9D Cell Culture

MN9D cells were purchased from American Type Culture Collection (ATCC, Manassas, VA, USA) and were cultured in Dulbecco's Modified Eagle Medium (GIBCO, Carlsbad, CA, USA) containing 8% fetal bovine serum (GIBCO, Carlsbad, CA, USA), 2 U mL^−1^ penicillin (Beyotime Biotechnology), and 2 mg mL^−1^ streptomycin (Beyotime Biotechnology) in an incubator at 37 °C and 5% CO_2_.

##### Cell Viability Assay

A MN9D cell suspension (≈1 × 10^4^ cells) was added to 96‐well plates. After treatment with DMC, nDMC, or RVG‐nDMC with or without 1000 × 10^−6^ m MPP^+^ for 48 h, CCK‐8 solution was added to each well, and the plates were incubated for 1.5 h. The absorbance at 450 nm was measured with a microplate reader (PerkinElmer). Data were obtained from three separate experiments, each of which were performed in triplicate.

##### Mitochondrial Superoxide Imaging of Live Cells

MitoSOX (Thermo Fisher Scientific) and Hoechst 33342 Staining (Beyotime Biotechnology) working solutions were added to MN9D cells. After incubation for 10 min at 37 °C, the cells were washed three times, and the images were scanned under a confocal laser‐scanning microscope (SP8; Leica).

##### ROS Detection Assay

2,7‐dichlorofluorescein diacetate (DCFH‐DA) (Beyotime Biotechnology) and Hoechst 33342 Staining (Beyotime Biotechnology) working solutions were added to MN9D cells. After incubation for 20 min at 37 °C, the cells were washed three times, and images were scanned under a confocal laser‐scanning microscope (SP8; Leica).

##### SOD Detection Assay

A Cu/Zn‐SOD Assay Kit with WST‐8 (Beyotime Institute of Biotechnology, Shanghai, China) was used to determine intracellular SOD activity. Briefly, MN9D cells were homogenized and incubated with WST‐8/enzyme working solution at 37 °C for 30 min. The absorbance was measured at a wavelength of 450 nm with a microplate reader (PerkinElmer). The protein concentrations were determined using BCA assay. The results were expressed as units per mg protein (U mg^−1^ protein).

##### GSH and GSSG Detection Assay

GSH and GSSG detection was performed using the GSH/GSSG kit (S0053, Beyotime Institute of Biotechnology, Shanghai, China) according to the manufacturer's instructions. Cells were collected and Protein Removal solution was added. The samples were then freeze‐thawed twice rapidly in liquid nitrogen and 37 °C water alternatively. After centrifugation at 10 000 × *g* for 10 min, the supernatant was used for the determination of total glutathione. GSH scavenging reagent working solution was added to degrade GSH. Then total glutathione working solution and NADPH solution were added to the supernatant to detect the concentration of total glutathione and GSSG. The GSH concentration was obtained from the total glutathione concentration minus the double GSSG concentration, and the GSH/GSSG ratio was determined. The absorbance at 412 nm was measured with a microplate reader (PerkinElmer). Data were obtained from three separate experiments, each of which were performed in triplicate.

##### NADPH/NADP^+^ Ratio Determination

The MN9D cellular NADPH/NADP^+^ ratio was measured using the NADPH/NADP^+^ assay kit (S0179, Beyotime Institute of Biotechnology, Shanghai, China) according to the manufacturer's instructions. Briefly, the samples were extracted from MN9D cells with extraction buffer and deproteinized with a spin column. Next, the extracted samples were resuspended in the NADP^+^/NADPH extraction buffer included in the NADP^+^/NADPH quantification kit. A series of standards were also prepared and measured colorimetrically, and the resulting standard curve was then used to calculate the ratio of NADPH/NADP^+^ in the samples. The absorbance at 450 nm was measured with a microplate reader (PerkinElmer). Data were obtained from three separate experiments, each of which were performed in triplicate.

##### RNA Sequencing Analysis

RNA sequencing was performed according to the previous report.^[^ ^46^ ^]^ Briefly, total RNA was extracted from nigral samples using Trizol (Life Technologies, Carlsbad, CA, USA). Next, RNA libraries were prepared using the NEBNext UltraTM RNA Library Prep Kit for Illumina (NEB, USA) according to the manufacturer's instructions. The qualities of purified libraries were assessed on the Agilent Bioanalyzer 2100 system. Clustering of the index‐coded samples was performed on a cBot Cluster Generation System using the TruSeq PE Cluster Kit v3‐cBot‐HS (Illumia), according to the manufacturer's instructions. After cluster generation, the samples were sequenced on an Illumina HiSeq 2500 platform (San Diego, CA). HTSeq v0.6.0 was used to count the read numbers mapped to each gene, and fragments per kilobase of transcript‐per‐million mapped reads (FPKM) of each gene were calculated based on the length of the gene and read counts mapped to the analyzed gene. DEG analysis was performed using the DESeq2 R package (1.10.1). The resulting *p*‐values were adjusted using the Benjamini‐Hochberg approach for controlling false discovery rates. The functional enrichment of DEGs was analyzed by the KEGG database.

##### Metabolomics Analysis of Nigral Samples

Nigral tissues were overlaid with 80% methanol solution containing 0.1% formic acid (FA) and vortexed. After centrifugation at 15 000 rpm, 4 °C for 10 min, the supernatant was collected for liquid chromatography‐tandem mass spectrometry (LC‐MS/MS) analysis. LC‐MS/MS analysis was performed using a Vanquish UHPLC system (Thermo Fisher) coupled with an Orbitrap Q Exactive series mass spectrometer (Thermo Fisher) as previously described.^[^ ^47^ ^]^ Samples were injected into a Hyperil Gold column (100 × 2.1 mm, 1.9 µm) using a 16 min linear gradient at a flow rate of 0.2 mL min^−1^. The Q Exactive series mass spectrometer was operated in positive/negative polarity mode with a spray voltage of 3.2 kV, capillary temperature of 320 °C, sheath gas flow rate of 35 arb, and aux gas flowrate of 10 arb. The metabolites data analysis were performed using the KEGG (http://www.genome.jp/kegg/), HMDB (http://www.hmdb.ca/) and Lipidmaps (http://www.lipidmaps.org/) databases, as previously described.^[^ ^47^ ^]^

##### TEM

The ultrastructural morphology of mitochondria was analyzed by TEM as described previously.^[^ ^48^ ^]^ Briefly, fixed tissues were dehydrated with an increasing gradient of alcohol and acetone, and then the tissues were embedded with 812 embedding agent (SPI‐Pon 812 Epoxy Resin Monomer; SPI, Shanxi, China). The sliced sections were double‐stained with uranium lead and dried overnight at RT, and the photographs were imaged and analyzed by transmission electron microscopy (HT7700; Hitachi, Tokyo, Japan).

##### Quantitative Analysis of Neurotransmitters

The nigral levels of DA, DOPAC, 5‐HT, and 5‐HIAA were determined using HPLC‐MS/MS. Nigral tissues were homogenized with methanol containing 0.1% FA, and supernatants were obtained after centrifugation at 14 000 rpm for 10 min. Then the samples were subjected to HPLC‐MS/MS analysis. HPLC‐MS/MS analysis was performed using UltiMate 3000 RS (Thermo Fisher) coupled with Q Exactive (Thermo Fisher). The samples were injected into a Waters T3 column (150 × 2.1 mm, 3 µm) at a flow rate of 0.3 mL min^−1^. The eluent for the aqueous phase mode was 0.1% FA in water; and the eluent for the organic phase was 0.1% ethyl formate. The Q Exactive series mass spectrometer was operated in positive/negative polarity mode with a spray voltage of 3.2 kV, a capillary temperature of 300 °C, and a sheath gas flow rate of 40 arb. The chromatogram acquisition and integration of each analyte were processed by Xcalibur 4.0 software (Thermo Fisher). The peak area of each analyte was taken as the ordinate and the concentration as the abscisic coordinate, and a standard curve was obtained by regression with the weighted coefficient. The sample concentration was calculated from the standard curve.

##### Western Blot Assay

Substantia nigra tissue was collected, and total protein was extracted with RIPA Lysis Buffer (Beyotime Biotechnology) and quantified using the BCA Protein Assay Kit (Beyotime Biotechnology). The samples were separated by SDS‐PAGE and transferred to PVDF membranes. Then the membranes were blocked with 5% BSA and incubated with primary antibody. After incubation with secondary antibody, chemiluminescence was visualized on the GeneGnome XRQ Chemiluminescence imaging system (Gene Company, Hong Kong, China). Image J software was used to analyze the optical density of bands.

##### Co‐Immunoprecipitation

Nigral tissues were lysed by IP lysis buffer, and then the lysates were centrifuged at 12 000 rpm at 4 °C for 10 min, and supernatant was collected. TH or Ub antibody was added to the supernatant, with IgG antibody as a negative control. After incubation at 4 °C overnight, HRP‐conjugated Protein A Sepharose beads were added to the supernatant, and the samples were incubated at 4 °C for 4 h and then boiled and subjected to western blot assay.

##### Immunohistochemistry and Immunofluorescence Assays

Embedded mouse brains were cut into 15 µm sections with a freezing microtome (Leica), and the slices were incubated with corresponding primary antibodies overnight at 4 °C. For the immunohistochemistry assay, brain slices were incubated with a secondary antibody labeled with biotin and stained with diaminobenzidine (DAB). Images were scanned under a microscope (Leica CS2, Hamburg, Germany). For the immunofluorescent assay, brain slices were incubated with fluorescent‐labeled secondary antibody, and DAPI was used to stain nuclei. Images were scanned under a confocal laser‐scanning microscope (SP8; Leica). Quantitative analysis was performed using the Image‐Pro Plus 6.0 photogram analysis system (IPP 6.0, Media Cybernetics, Bethesda, MD, USA).

##### Quantitative RT‐PCR

Total RNA from tissues was extracted in Trizol (Invitrogen, San Diego, CA, USA). The genomic DNA was removed by using DNA Eraser Buffer (Takara, Otsu, Japan). RNA was reverse transcribed into cDNA, and qPCR was performed with TB Green Premix Ex Taq (Takara). The thermal parameters were as follows: 95 °C for 5 s, 55 °C for 30 s, and 72 °C for 30 s by 40 cycles. The primer sequences were as follows: IL‐1*β* forward 5’‐ AATGCCACCTTTTGACAGTGAT‐3’ and reverse 5’‐ TGCTGCGAGATTTGAAGCTG‐3’; TNF‐*α* forward 5’‐ CACGTCGTAGCAAACCACC‐3’ and reverse 5’‐ TGAGATCCATGCCGTTGGC‐3’; IL‐6 forward 5’‐AGGATACCACTCCCAACAGACC‐3’ and reverse 5’‐AAGTGCATCATCGTTCATACA‐3’; GAPDH forward 5’‐ACGGGAAGCTCACTGGCATGGCCTT‐3’ and reverse 5’‐CATGAGGTCCACCACCCTGTTGCTG‐3’.

##### Enzyme‐Linked Immunosorbent Assay (ELISA)

Mouse nigral and striatal IL‐1*β*, TNF‐*α*, and IL‐6 concentrations were measured using ELISA kits (Shanghai Enzyme‐linked Biotechnology Co., Ltd., Shanghai, China) as described in a previous study.^[^ ^47^ ^]^ Briefly, nigral and striatal samples were homogenized in ice‐cold PBS containing protease inhibitors, and the homogenates were then centrifuged to obtain the supernatants. The resulting samples were incubated with the indicated antibody‐coated plates and horseradish peroxidase (HRP)‐labeled secondary antibody at 37 °C for 1 h. After incubation with chromogenic agents A and B at 37 °C for 15 min, the OD values were measured by Multiscan Spectrum (PerkinElmer, MA, USA) at 450 nm. The protein concentrations were determined using BCA assay. The results were expressed as pg per mg protein (pg mg^−1^ protein).

##### Multianalyte Suspension Arrays Application

Each sample in 96‐well microtiter plates was incubated with bead mixture (100 microspheres with type‐specific probes in 25 µL) for 2 h at RT, and subsequently with denatured biotin‐labeled detective antibody complex (50 µL) for 1 h at RT. After three washes, the samples with microspheres were incubated with streptavidin‐labelled phycoerythrin (50 µL) for 30 min at RT in the dark. Finally, the reaction product was detected by a Luminex 100 analyzer instrument (Luminex Corporation, Austin, USA). Each reaction was repeated in triplicate.

##### Statistical Analysis

Data are presented as mean ± standard error of the mean (SEM). For behavioral experiments, 9–10 per group were used for measurement. For in vitro immunofluorescence experiments, 6 per group were used for measurement. For in vivo immunofluorescence, immunohistochemistry, metabolomic analysis, and western blot experiments, 6 per group were used for measurement. For ultrastructural analysis using TEM, 10 per group were used for measurement. Data were analyzed using the unpaired Student's *t*‐test or one‐way ANOVA followed by a Tukey's post‐hoc test, where appropriate. The Hotelling T2 test was used to evaluate outliers in the metabolomic analysis. Differences with a *p*‐value < 0.05 were considered statistically significant. All statistical analyses were performed using GraphPad Prism 8.0 (GraphPad Software, La Jolla, CA). Representation of the *p*‐value was ^*^ *p* < 0.05, ^**^ *p* < 0.01.

## Conflict of Interest

The authors declare no conflict of interest.

## Supporting information

Supporting Information

## Data Availability

The data that supports the findings of this study are available in the supplementary material of this article.
