# Supplementary material for: Trojan Horse Delivery of 4,4′‐Dimethoxychalcone for Parkinsonian Neuroprotection
Source: Adv Sci (Weinh). 2021 Mar 3;8(9):2004555. doi: 10.1002/advs.202004555 (PMC8097374; doi:10.1002/advs.202004555)
Supplement: Supplementary file 1 — Supporting Information [file ADVS-8-2004555-s001.pdf]

© 2021 Wiley-VCH GmbH

ADVANCED SCIENCE
Open Access

Supporting Information

for *Adv. Sci.*, DOI: 10.1002/advs.202004555

Trojan Horse Delivery of 4,4′-Dimethoxychalcone for Parkinsonian Neuroprotection

*Wenlong Zhang, Huaqing Chen, Liuyan Ding, Junwei Gong, Mengran Zhang, Wenyuan Guo, Pingyi Xu, * Shiying Li, * Yunlong Zhang **

Supporting Information

Trojan Horse Delivery of 4,4′-Dimethoxychalcone for Parkinsonian Neuroprotection

*Wenlong Zhang, Huaqing Chen, Liuyan Ding, Junwei Gong, Mengran Zhang, Wenyuan Guo, Pingyi Xu, * Shiying Li, * Yunlong Zhang **

J. Gong, M. Zhang, Prof. Y. Zhang

Key Laboratory of Neurological Function and Health,

School of Basic Medical Sciences,

Guangzhou Medical University,

Guangzhou 511436, China.

E-mail: ylzhang@gzhmu.edu.cn

H. Chen and Prof. S. Li

Key Laboratory of Molecular Target & Clinical Pharmacology and the State Key Laboratory of Respiratory Disease,

School of Pharmaceutical Sciences & The Fifth Affiliated Hospital,

Guangzhou Medical University,

Guangzhou 511436, China.

E-mail: lisy-sci@gzhmu.edu.cn

W. Zhang, L. Ding, W. Guo, Dr. P. Xu

Department of Neurology,

The First Affiliated Hospital of Guangzhou Medical University,

Guangzhou 510120, China.

E-mail: pingyixu@sina.com

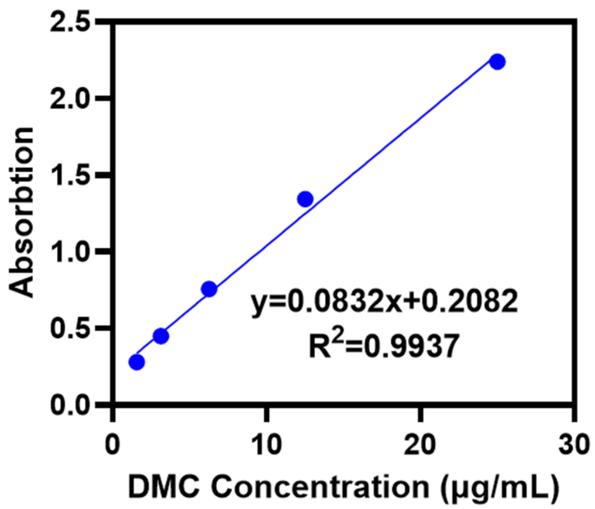

Figure S1. Characterization of nDMC and RVG-nDMC. Standard curve of UV-vis absorbance of DMC at 350 nm used to measure the drug encapsulation efficiency (EE), loading efficiency (LE) and release behaviors of nDMC and RVG-nDMC.

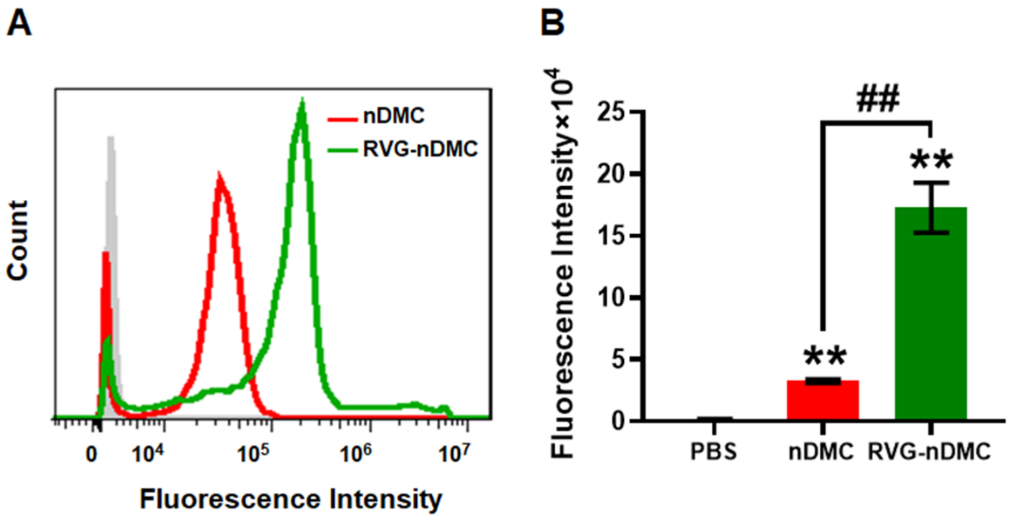

Figure S2. *In vitro* cellular uptake of nanoparticles. Flow cytometry was employed to quantitatively analyze difference in the uptake of nDMC and RVG-nDMC. In brief, MN9D cells were seeded at a density of 1×10^6 cells/well into 6-well plates. After 24 h incubation, the culture medium was replaced with fresh medium containing nDMC/RVG-nDMC (2 μg/mL of DMC, 2 μg/mL of Cy5.5). After another 6 h incubation, the cells were washed with PBS three times and collected for flow cytometry analysis (Ex: 638 nm, Em: 712/25 nm) (Beckman Cytoflex, USA). n = 3 per group. Results are expressed as the mean ± SEM. $^{**}p < 0.01$. $^{\#\#}p < 0.01$ vs. nDMC group. One-way ANOVA with Tukey *post-hoc* analysis was used for comparison among multiple groups.

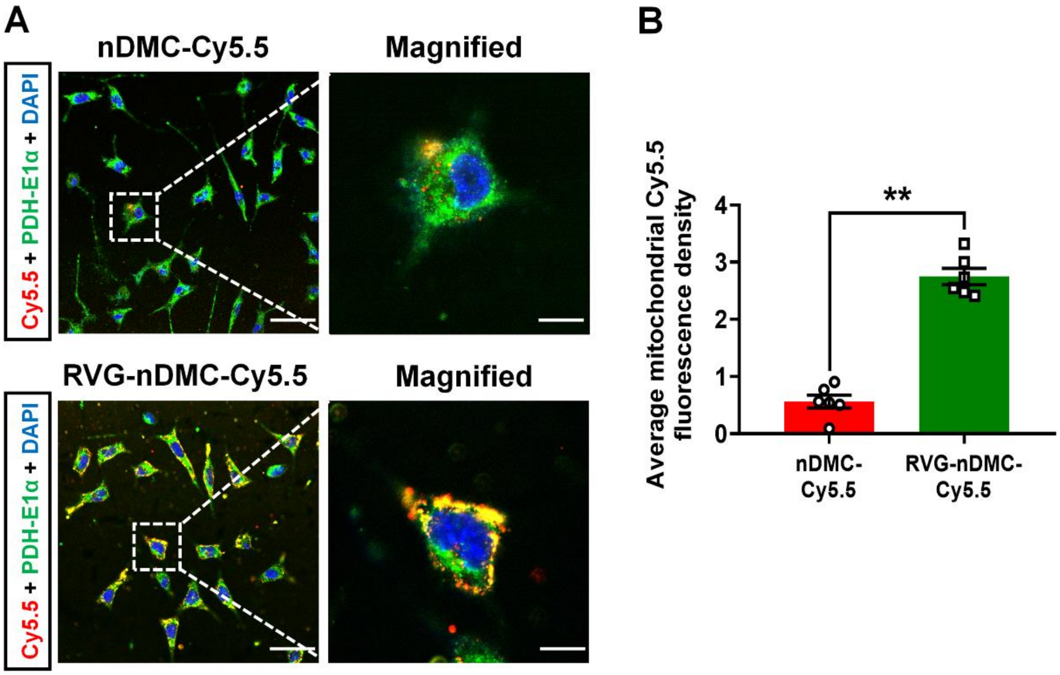

Figure S3. Distribution of Cy5.5-loaded nanoparticles in mitochondria. MN9D cells were incubated with Cy5.5-labelled nDMC and RVG-nDMC for 6 h, and then the images were obtained using confocal laser-scanning microscope. Representative images (A) and quantitative fluorescence analysis of mitochondrial marker (PDH-E1α) in MN9D cells (B) were shown. n = 6 in each group. Results are expressed as the mean ± SEM. $^{**}p < 0.01$. Unpaired Student's *t*-test was used for comparison between two groups.

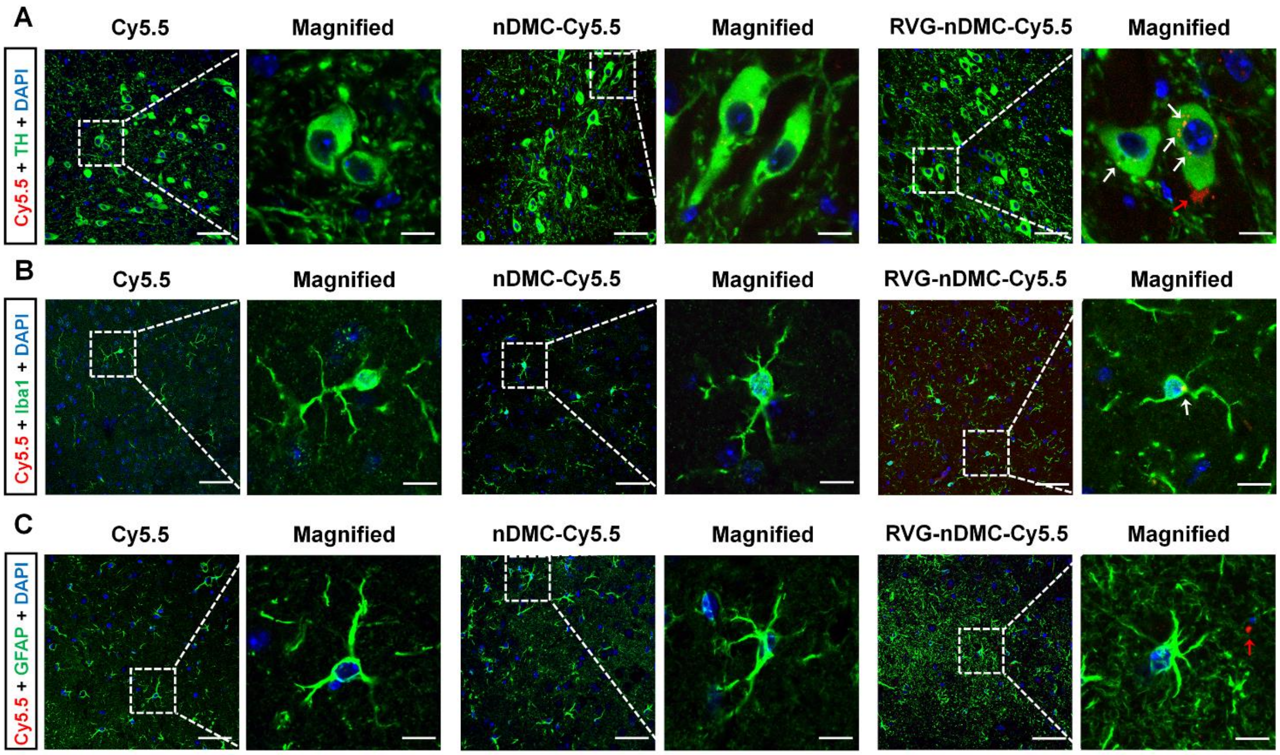

Figure S4. ***In vivo*** **images of RVG-nDMC nanoparticles administration for 1 h in the SNpc of normal mice.** (A-C) Representative images of TH, Iba1 and GFAP staining in the SN derived from mice treated with Cy5.5, nDMC-Cy5.5 and RVG-nDMC-Cy5.5 1 h post-injection. White arrows in the enlarged details of the right column show the presence of nanoparticles in the DA neuron and microglia. Red arrows in the enlarged details of the right column show the presence of nanoparticles outside of DA neuron and astrocytes. Scale bars, 50 μm for the original images and 10 μm for the magnified images.

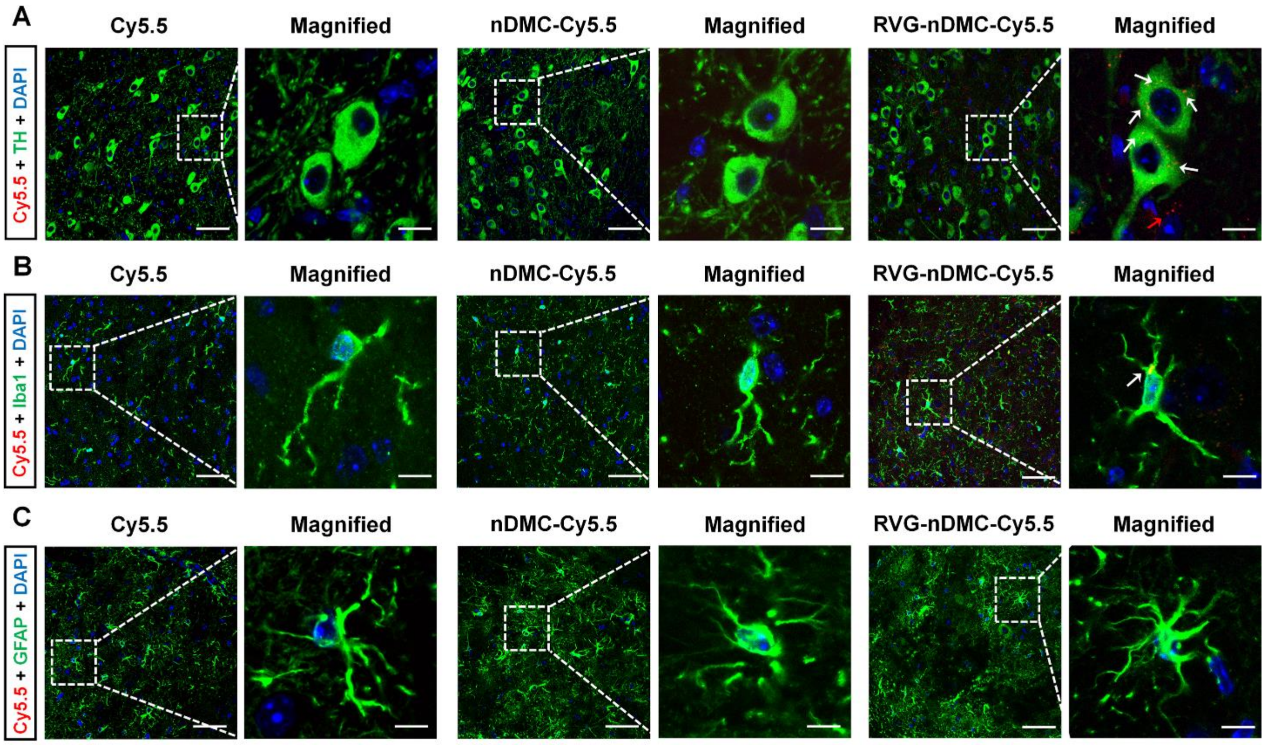

Figure S5. *In vivo* images of RVG-nDMC nanoparticles administration for 24 h in the SNpc of normal mice. (A-C) Representative images of TH, Iba1 and GFAP staining in the SN derived from mice treated with Cy5.5, nDMC-Cy5.5 and RVG-nDMC-Cy5.5 24 h post-injection. White arrows in the enlarged details of the right column show the presence of nanoparticles in the DA neuron and microglia. Red arrows show the presence of nanoparticles outside of DA neuron. Scale bars, 50 μm for the original images and 10 μm for the magnified images.

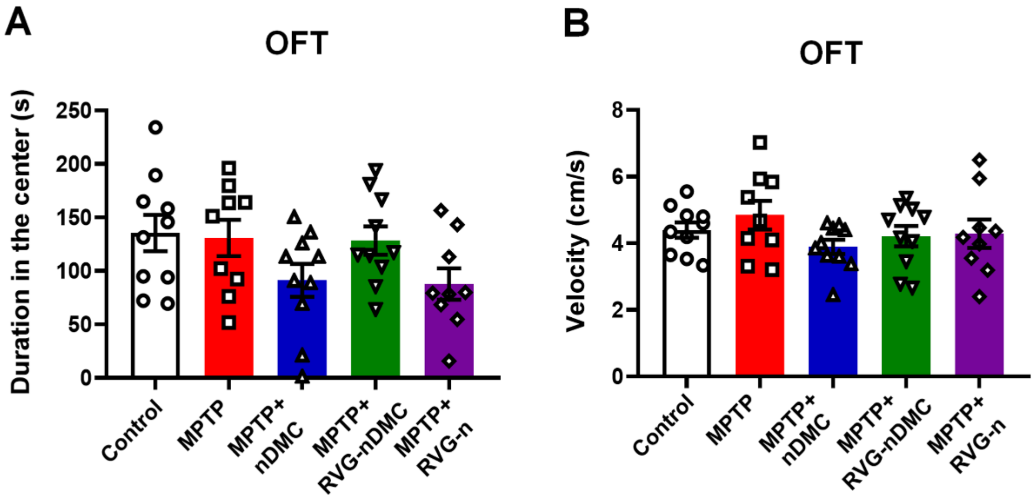

Figure S6. Behavioral performance of RVG-nDMC treatment in the PD model in the open field test. (A and B) Time spent in the center and movement speed in the open-field of MPTP-induced PD mice treated with nDMC, RVG-nDMC and RVG-n. n = 10, 9, 10, 10, and 9 for Control, MPTP, MPTP + nDMC, MPTP + RVG-nDMC and MPTP + RVG-n groups, respectively. Results are expressed as the mean ± SEM. Statistical significance was determined by one-way ANOVA and Tukey tests for *post-hoc* comparisons.

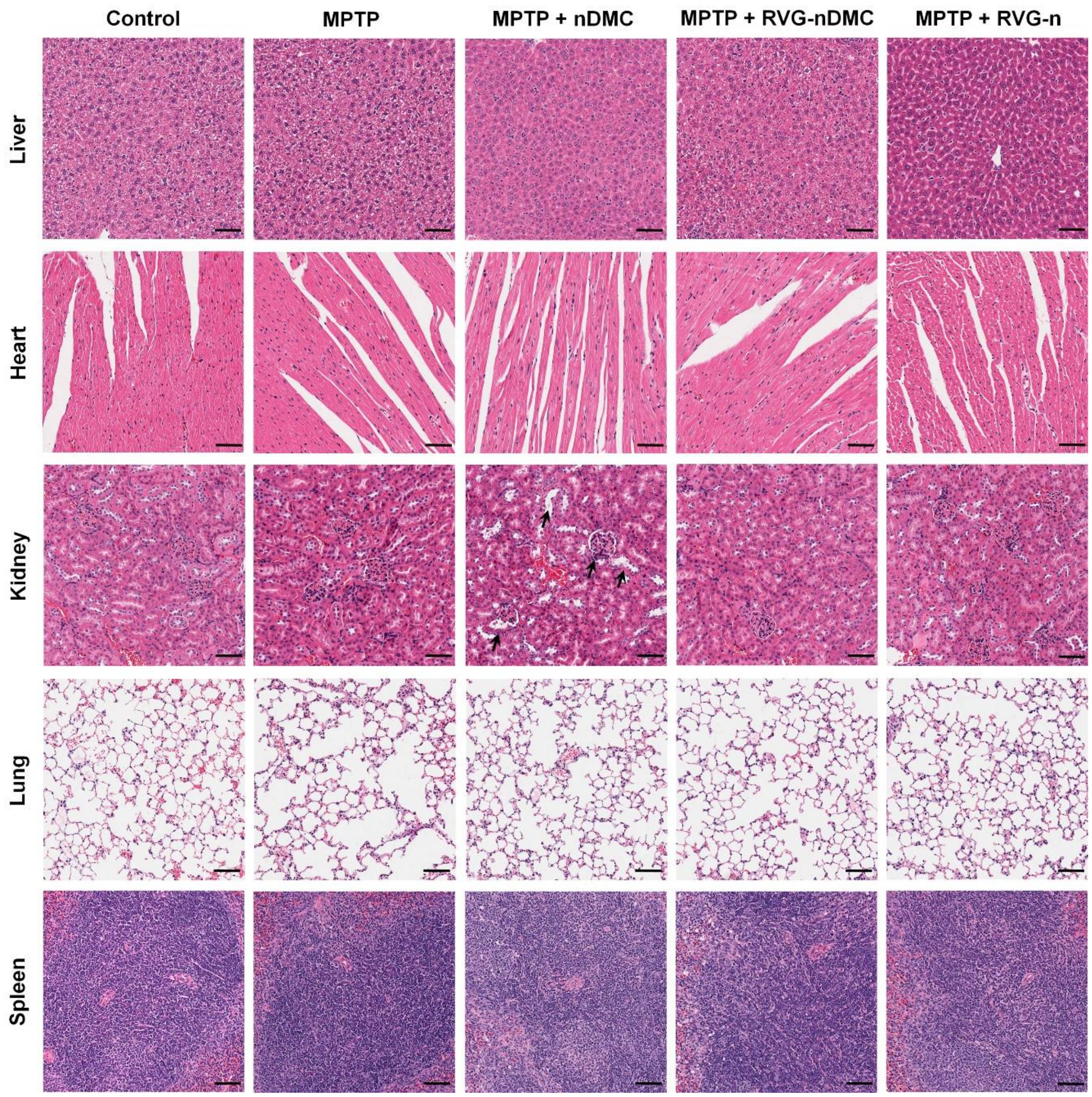

Figure S7. RVG-nDMC exerted no observable toxicity in major organs. Representative graphs for hematoxylin and eosin staining in major organs (including liver, heart, kidney, lung and spleen) from Control, MPTP, MPTP + nDMC, MPTP + RVG-nDMC and MPTP + RVG-n groups, n = 3 per group. There were no detectable pathological changes in the major organs derived from MPTP mice treated with RVG-nDMC nanoparticles. However, nDMC resulted in observable renal tubular atrophy and interstitial loosening in the kidney. Black arrows indicate pathological injury. Scale bars, 100 µm.

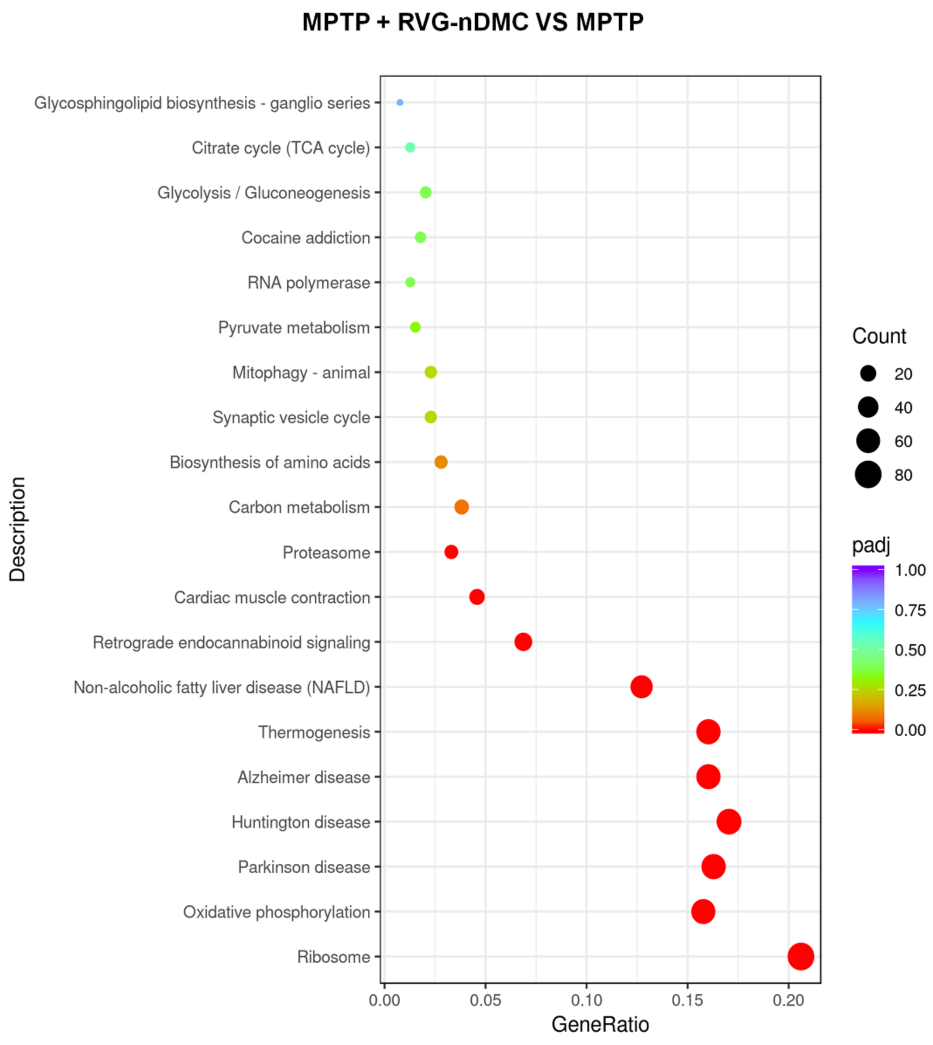

Figure S8. KEGG analysis between MPTP + RVG-nDMC and MPTP group. KEGG pathways enriched by DEGs derived from RNA-sequencing between MPTP + RVG-nDMC and MPTP groups.

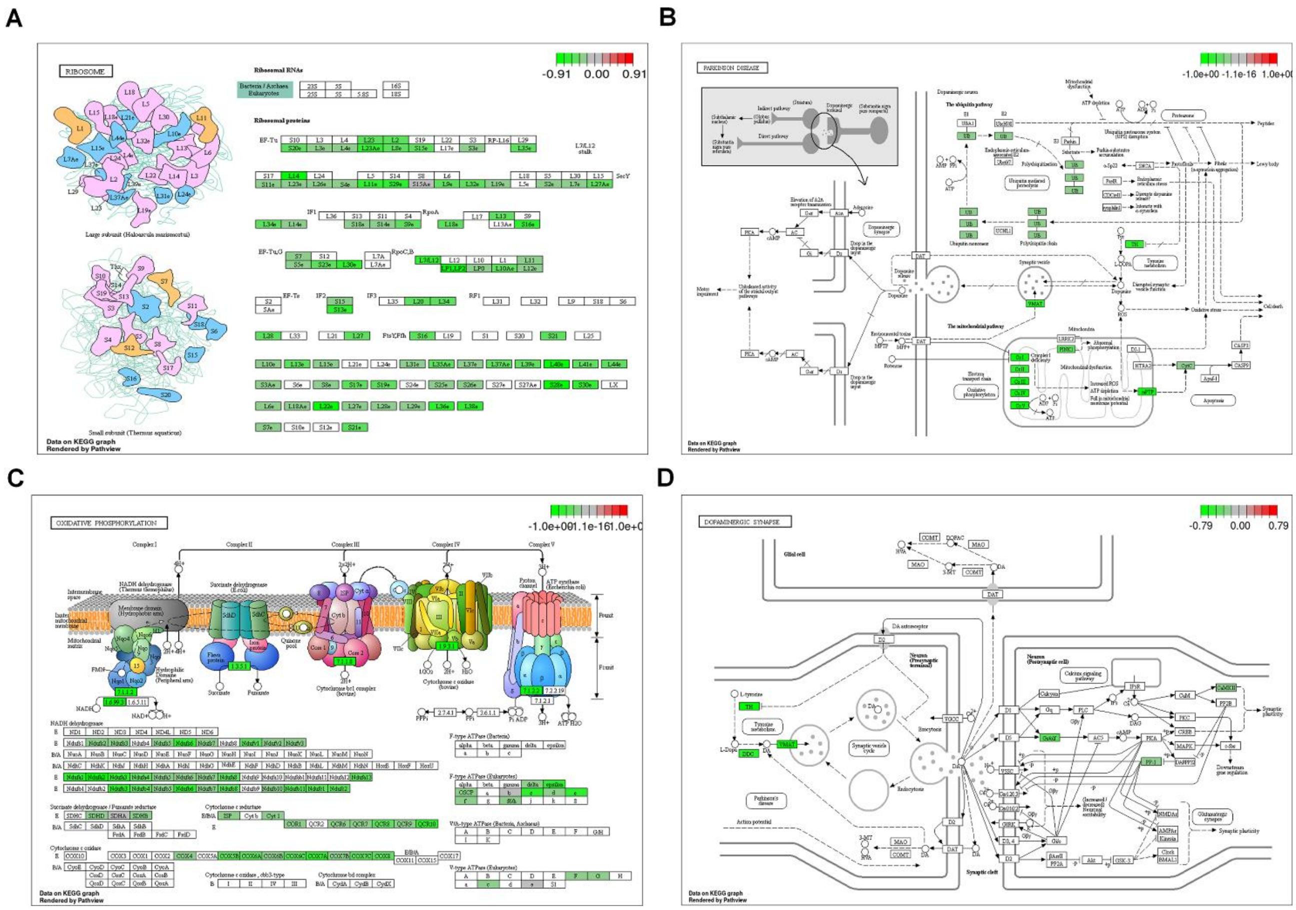

Figure S9. Distribution of the DEGs in selected KEGG pathways. (A-D) the altered genes among the three groups derived from RNA-sequencing are colored green for Ribosome (A), Parkinson's disease (B), Oxidative phosphorylation (C) and Dopaminergic synapse (D) pathways.

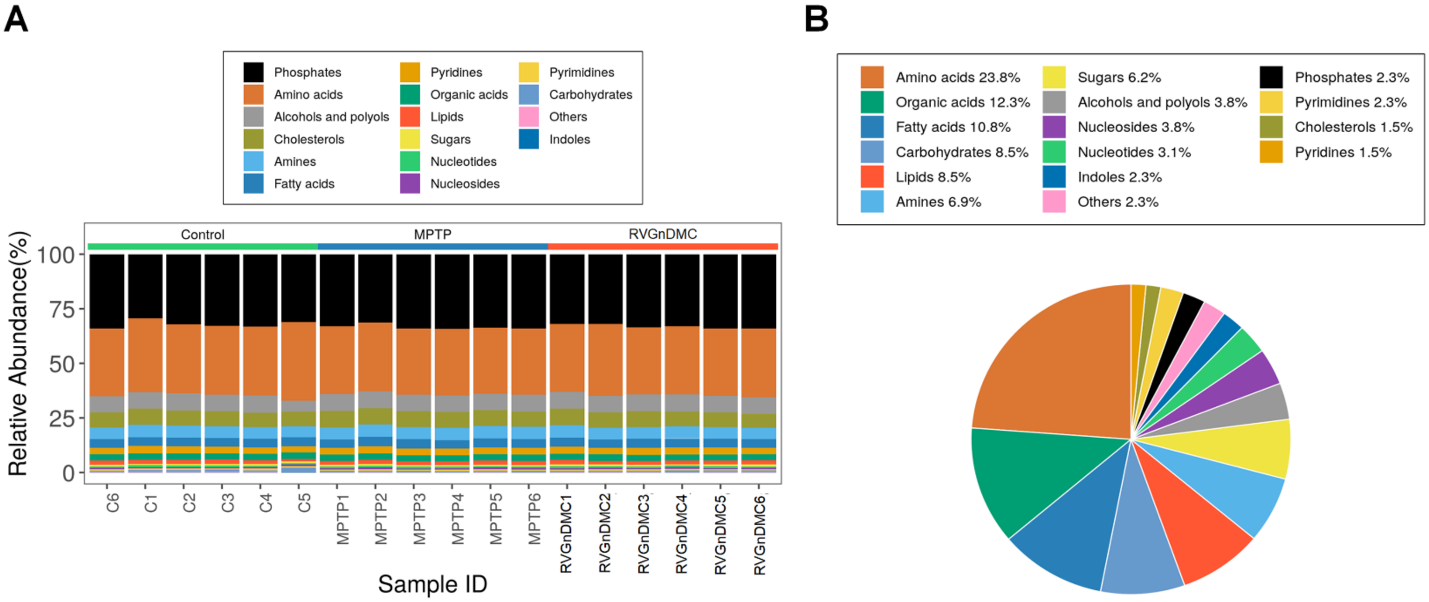

Figure S10. Metabolomic profiling of the nigral metabolites upon RVG-nDMC treatment in MPTP-treated mice. (A) The relative abundances of representative metabolites in metabolomic analysis in each sample are shown. (B) The annotated metabolites and their chemical classes in the metabolomic analysis are illustrated.

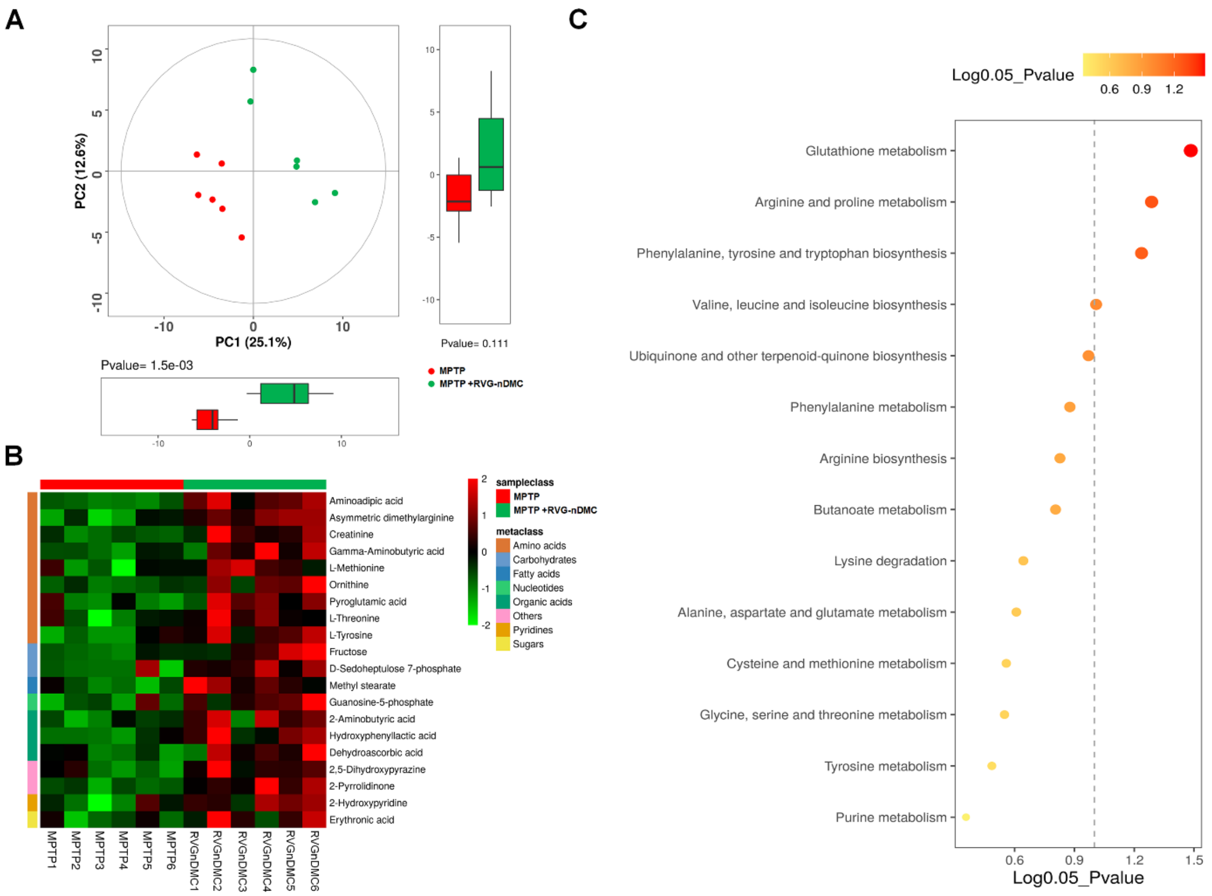

Figure S11. Metabolomic analysis of the nigral metabolites between MPTP and MPTP + RVG-nDMC groups. (A) Overall metabolic profiles of samples from the MPTP and MPTP + RVG-nDMC groups using PCA plots. (B) Z-score plots showing the relative variations of each individual metabolite between these two groups in the form of a heatmap. (C) MPEA of potential pathways involved in the differential metabolite content between these two groups.

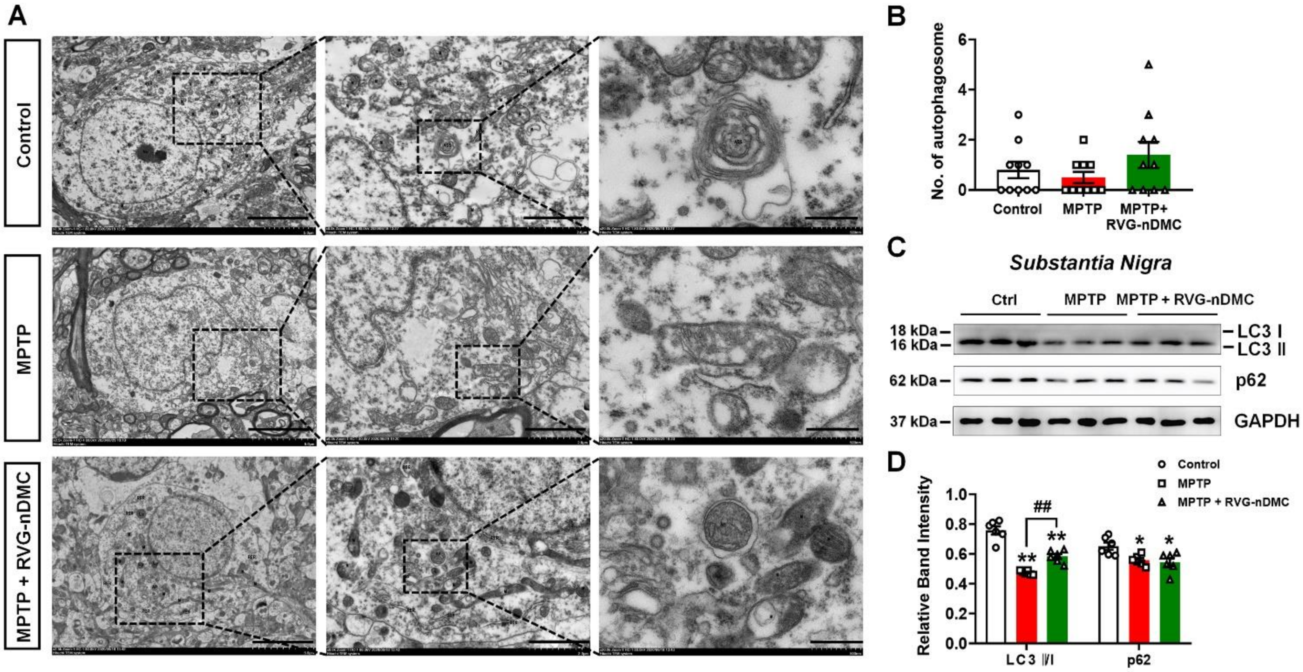

Figure S12. Effects of RVG-nDMC treatment on the autophagy flux in PD mice. (A) Ultrastructural analysis of autophagosomes in the SN of Control, MPTP and MPTP + RVG-nDMC groups. Scale bars, left, 5 μm; middle, 2 μm; right, 500 nm. (B) Quantification of the number of autophagosomes in the three groups, n = 10 per group. (C and D) Representative blots and quantification showing the LC3 Ⅱ/I and p62 expression levels in the SN of three groups, n = 6 per group. Results are expressed as the mean ± SEM. $^{**}p < 0.01$, $^{*}p < 0.05$ vs. Control. $^{\#\#}p < 0.01$ vs. MPTP group. Statistical significance was determined by one-way ANOVA and Tukey tests for *post-hoc* comparisons.

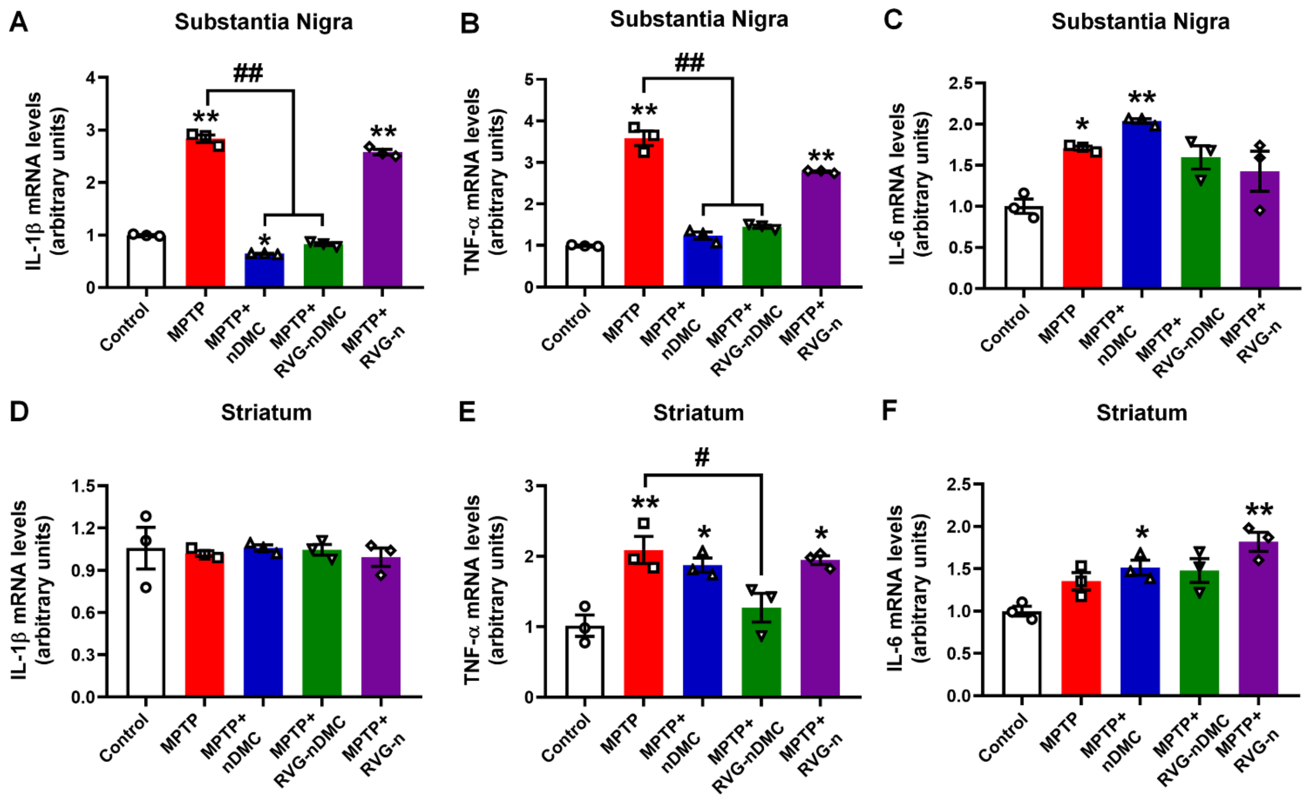

Figure S13. RVG-nDMC attenuates neuroinflammation in SN and striatum of PD mice. Quantitative analysis of IL-1β, TNF-α and IL-6 mRNA expression in SN (A-C) and striatum (D-F) upon treatment with different nanoparticles in MPTP mice, n = 3 per group. Results are expressed as the mean ± SEM. $^{**}p < 0.01$, $^{*}p < 0.05$ vs. Control. $^{\#\#}p < 0.01$, $^{\#}p < 0.05$ vs. MPTP group. Statistical significance was determined by one-way ANOVA and Tukey tests for *post-hoc* comparisons.

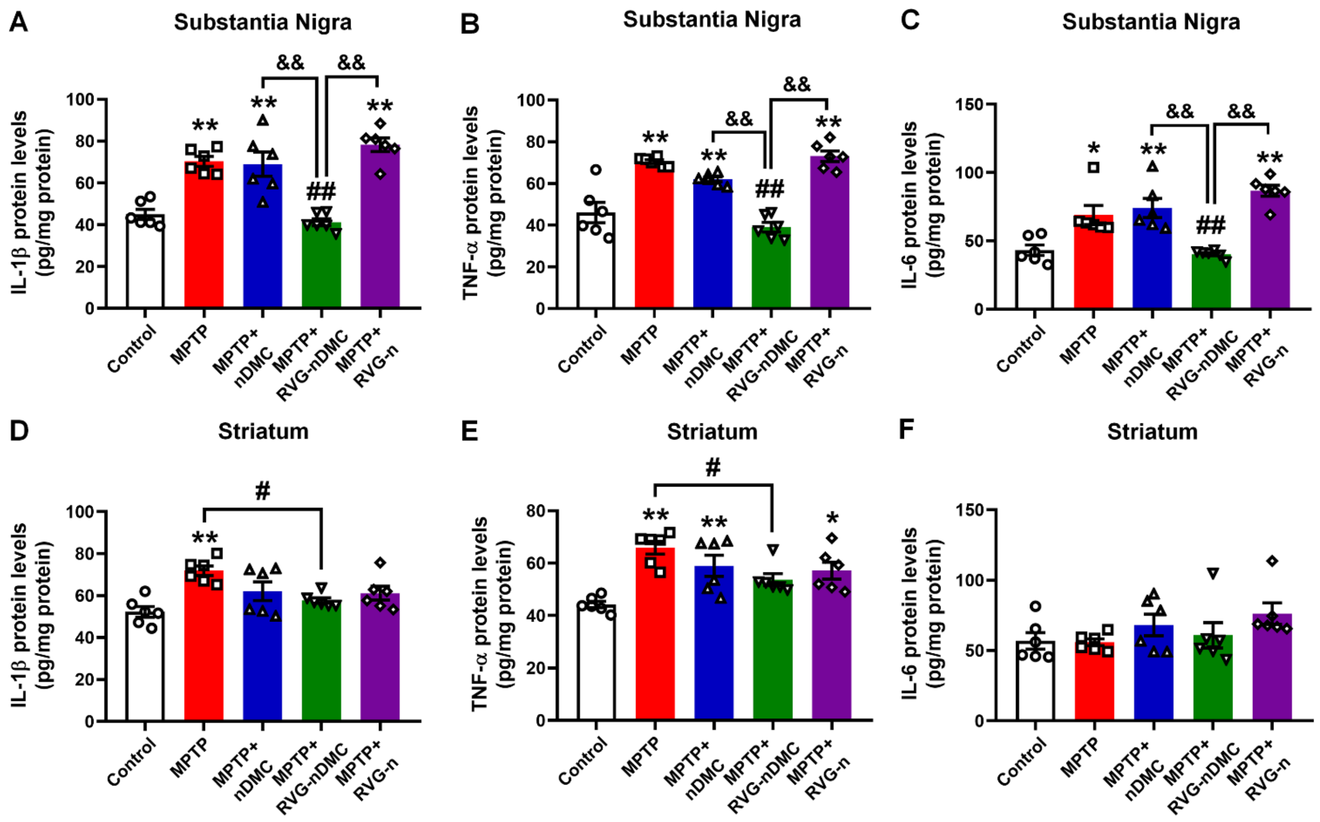

Figure S14. RVG-nDMC reduced the protein levels of pro-inflammatory cytokines in SN and striatum of PD mice. Quantitative analysis of IL-1β, TNF-α and IL-6 protein expression in SN (A-C) and striatum (D-F) upon treatment with different nanoparticles in MPTP mice using ELISA, n = 6 per group. Results are expressed as the mean ± SEM. $^{**}p < 0.01$, $^{*}p < 0.05$ vs. Control. $^{\#\#}p < 0.01$, $^{\#}p < 0.05$ vs. MPTP group. $^{\&\&}p < 0.01$ vs. MPTP + RVG-nDMC group. Statistical significance was determined by one-way ANOVA and Tukey tests for *post-hoc* comparisons.

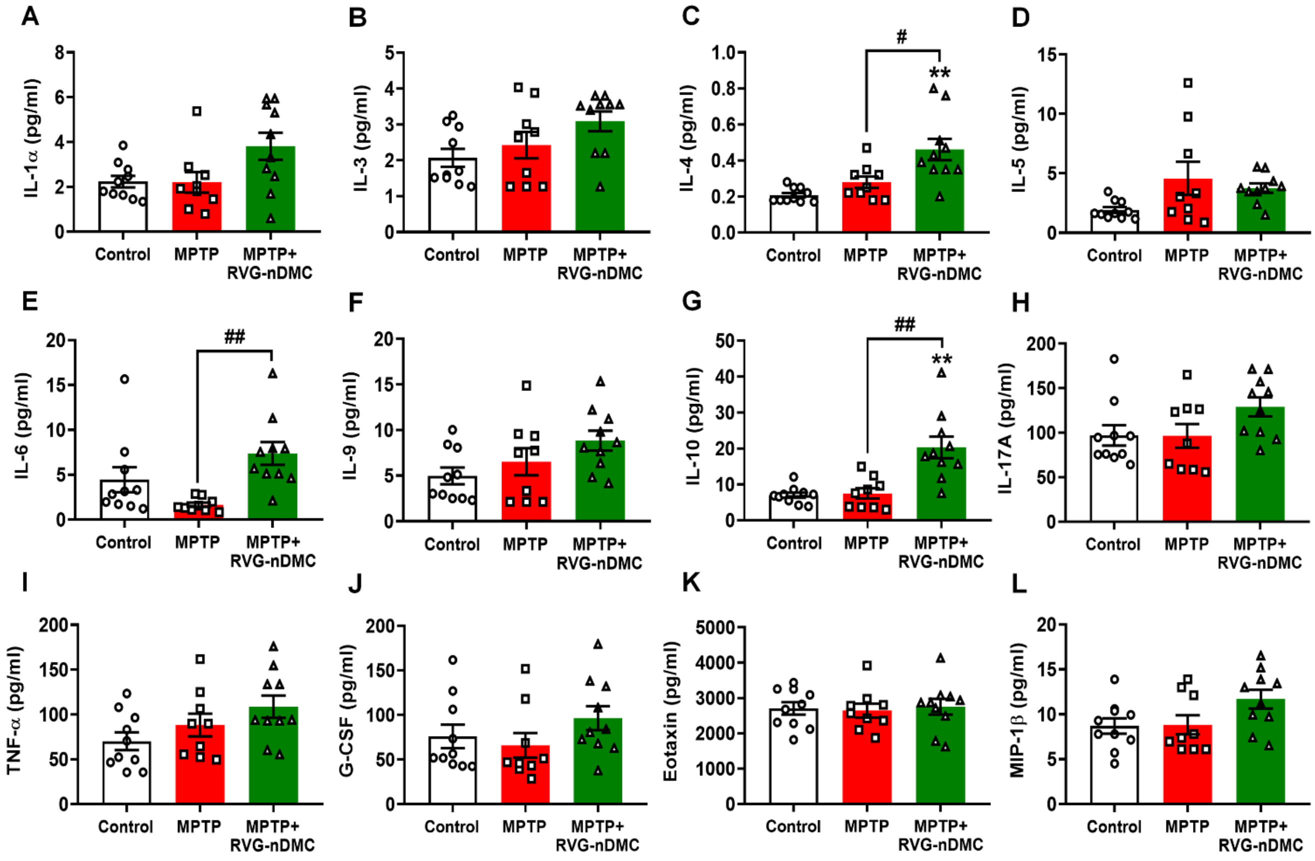

Figure S15. Effect of RVG-nDMC administration on serum inflammatory cytokine expression in PD mice. Quantitative analysis of IL-1α (A), IL-3 (B), IL-4 (C), IL-5 (D), IL-6 (E), IL-9 (F), IL-10 (G), IL-17A (H), TNF-α (I), G-CSF (J), Eotaxin (K), and MIP-1β (L) in the serum upon treatment with RVG-nDMC in MPTP mice, n = 10, 9, 10 in Control, MPTP and MPTP + RVG-nDMC groups, respectively. Results are expressed as the mean ± SEM. $^{**}p < 0.01$ vs. Control. $^{\#\#}p < 0.01$, $^{\#}p < 0.05$ vs. MPTP group. Statistical significance was determined by one-way ANOVA and Tukey tests for *post-hoc* comparisons.

Table S1. Analysis of liver and kidney function following nDMC, RVG-nDMC and RVG-n administration in MPTP mice.

	Control (n=3)	MPTP (n=3)	MPTP + nDMC (n=3)	MPTP + RVG-nDMC (n=3)	MPTP + RVG-n (n=3)	*P* Value
AST	121.00 (47.62)	111.67 (12.66)	91.00 (35.04)	154.00 (10.44)	96.67 (17.67)	0.132
ALT	51.33 (30.40)	31.67 (6.81)	37.67 (13.61)	75.00 (22.52)	32.67 (6.66)	0.076
AST/ALT	2.52 (0.46)	3.59 (0.50)	2.41 (0.39)	2.19 (0.75)	3.00 (0.50)	0.057
TP	54.07 (4.75)	53.20 (1.75)	52.13 (1.55)	51.9 0 (2.10)	53.13 (0.76)	0.838
ALB	21.60 (3.25)	21.40 (0.36)	19.53 (2.12)	19.77 (1.01)	20.70 (0.72)	0.536
GLOB	32.47 (1.50)	31.8 (1.40)	32.77 (1.55)	32.13 (1.11)	32.43 (0.78)	0.91
A/G	0.67 (0.06)	0.70 (0.03)	0.57 (0.06)	0.60 (0.05)	0.63 (0.06)	0.031
ALP	77.67 (2.89)	108.67 (14.22)	82.00 (29.72)	104.67 (25.15)	72.67 (6.43)	0.132
GLU	5.30 (1.40)	5.84 (0.89)	6.86 (1.63)	6.28 (1.15)	5.93 (1.42)	0.689
UREA	6.58 (0.74)	7.67 (0.83)	8.07 (0.08)	6.13 (0.37)	7.87 (1.43)	0.062
CR	11.33 (4.73)	11.00 (1.73)	14.00 (1.00)	9.00 (1.00)	14.67 (3.06)	0.142
UA	293.00 (47.15)	257.00 (34.60)	337.67 (88.87)	393.67 (25.58)	284.33 (30.24)	0.056
LDH	925.00 (110.01)	799.00 (158.62)	471.67 (128.49)	875.67 (209.35)	450.67 (85.56)	0.005

AST: aspartate aminotransferase; ALT: alanine aminotransferase; AST/ALT: aspartate aminotransferase alanine aminotransferase ratio; TP: Total protein; ALB: albumin; GLOB: globulin; A/G: albumin globulin ratio; ALP: alkaline phosphatase; GLU: blood glucose: UREA: urea nitrogen; CR: creatinine; UA: uric acid; LDH: lactate dehydrogenase. Results are expressed as the mean ± SEM. Statistical significance was determined by one-way ANOVA and Tukey tests for *post-hoc* comparisons.
